# Association of liver biomarker values beyond current thresholds and negative clinical outcomes in primary biliary cholangitis: analysis of a real-world healthcare claims database

**DOI:** 10.57264/cer-2024-0198

**Published:** 2025-11-18

**Authors:** Timothy E Ritter, Christina J Hanson, Christopher Fernandes, Joanna P MacEwan, Xin Zhao, Craig S Parzynski, Daniel Mercer, Erik Ness, Darren Wheeler, Kris V Kowdley, Tracy Mayne

**Affiliations:** 1 GI Alliance Research, 620 E Southlake Blvd, Southlake, TX 76092, USA; 2 South Denver Gastroenterology, 499 E Hampden Ave #420, Englewood, CO 80113, USA; 3 Stanford Medicine, Emergency Medicine, 900 Welch Road, Suite 350, Palo Alto, CA 94304, USA; 4 Genesis Research Group, 111 River St Ste 1120, Hoboken, NJ 07030, USA; 5 Intercept Pharmaceuticals, 305 Madison Ave, Morristown, NJ 07960, USA; 6 Liver Institute Northwest & Elson S. Floyd College of Medicine, Washington State University, 3216 NE 45th Place #212, Seattle, WA 98105, USA

**Keywords:** biochemical markers, clinical outcomes, primary biliary cholangitis, real-world data, treatment guidelines

## Abstract

**Aim:**

To assess the predictive effect of threshold deviations for multiple liver biomarkers on negative clinical outcomes, including hepatic decompensation, liver transplantation, and death in patients with primary biliary cholangitis (PBC) using longitudinal data from a large administrative claims database.

**Materials & methods:**

A time-dependent Cox proportional hazards model with time-dependent covariates assessed time to first occurrence of hospitalization for hepatic decompensation, liver transplantation, or death in patients with PBC in the Optum Clinformatics Data Mart™ database. Separate models analyzed proportion of time outside prespecified biomarker thresholds (defined as multiples of upper limit of normal [ULN] for alkaline phosphatase [ALP], total bilirubin [TB], aspartate aminotransferase [AST], and alanine aminotransferase [ALT]; lower limit of normal for albumin). Another model evaluated both ALP and TB with the lowest relevant threshold applied for each biomarker.

**Results:**

Overall, 2402 patients were included; 85.3% were female, mean age was 63.3 years, median follow-up was 2.2 years (interquartile range: 1.1–3.9 years). On average, patients had approximately five reported measurements for each biomarker evaluated. Each 10% increase in time outside thresholds was associated with a 6.5%, 9.2%, 9.9%, 7.3% and 23.3% increase in risk of negative outcomes for ALP, TB, AST, ALT and albumin, respectively.

**Conclusion:**

In patients with PBC, values outside predefined thresholds for biomarkers including ALP, TB, AST, ALT and albumin strongly predicted negative clinical outcomes. These findings highlight the importance of managing biomarkers beyond ALP in monitoring and the treatment of patients with PBC.

## Plain language summary: How abnormal liver test results are linked to worse health outcomes in patients with primary biliary cholangitis: a study using real-world health data

**Why was this study done?:**

Patients with primary biliary cholangitis are at risk of serious health problems if the disease is not treated properly. Certain abnormal liver test results at diagnosis or shortly after starting treatment can help predict these risks. One key liver test often used is alkaline phosphatase. However, it is not known whether regularly tracking liver tests over time or including tests in addition to alkaline phosphatase (aspartate aminotransferase, alanine aminotransferase, total bilirubin and albumin) can better predict who will develop these serious outcomes.

**What did this study look at?:**

This study used information from health insurance claims to look at whether abnormal liver test results, tracked over time, were linked to negative health problems such as hospitalization for serious liver complications (hepatic decompensation), liver transplantation or death in patients with primary biliary cholangitis.

**What were the results?:**

Patients who spent more time with abnormal liver test values had a greater risk of serious health problems. The longer they spent outside the normal range, the higher the risk of complications.

**What do the results mean?:**

It is important to monitor liver test results over time. In addition to ALP, other tests like aspartate aminotransferase, alanine aminotransferase, total bilirubin and albumin also play an important role in evaluating risk.

Primary biliary cholangitis (PBC) is a rare chronic liver disease characterized by the gradual destruction of the intrahepatic bile ducts. Published estimates of PBC prevalence in the US have varied between 16 and 40.2 per 100,000 persons [1–3]. A recent retrospective claims database analysis reported an adjusted 2021 estimated prevalence of 40.9 per 100,000 adults, indicating 105,506 adults with PBC in the US [4]. PBC is most prevalent in women aged older than 50 years, but male sex, younger age at onset (<45 years), and advanced disease at presentation are predictive of poorer outcomes [5]. Without sufficiently effective treatment, PBC may progress to cirrhosis and lead to negative clinical outcomes, including hepatic decompensation, liver transplantation and death [6,7].

A substantial proportion of patients have an inadequate response to ursodeoxycholic acid (UDCA), the only approved first-line therapy for PBC, and remain at risk for poor outcomes. However, the criteria for defining inadequate treatment response vary, which may hinder timely treatment decisions. For example, a previous study that used the Paris-I criteria of serum bilirubin ≤1 mg/dl, alkaline phosphatase (ALP) ≤3× the upper limit of normal (ULN), and aspartate aminotransferase (AST) ≤2 × ULN) found that approximately 40% of patients receiving first-line UDCA had an inadequate treatment response [8]. Another study, which used the Toronto criteria of reduction in ALP to <1.67 × ULN by 2 years of UCDA treatment, revealed a nonresponder rate of 24% [9].

A real-world retrospective study that evaluated multiple response criteria revealed a wide range of inadequate UCDA response rates at 12 months within the patient cohort: 9% with Paris-I criteria, 17% with ALP ≤1.67 × ULN, and 39% with the Barcelona criteria of ALP ≤1 × ULN or ALP reduction >40% [10]. Biochemical response rates among these patients improved with treatment modifications, including the initiation of second-line therapy [10]. Results of a more recent study suggested that an ALP level of 1.9 × ULN at 6 months of UCDA treatment is an indicator for initiating second-line therapy, introducing a higher ALP threshold as well as an earlier time point for treatment modification [11]. These studies reflect that treatment guidelines and clinical practice have largely depended on monitoring ALP to determine first-line treatment response in patients with PBC. Furthermore, they highlight the lack of uniform thresholds and timeframes for guiding treatment decisions [12,13].

Current clinical guidelines also focus on ALP thresholds as key indicators of negative clinical outcomes. The European Association for the Study of the Liver guidelines identified ALP >1.50 × ULN as the threshold for increased risk, while the American Association for the Study of Liver Diseases (AASLD) guidelines set the threshold at ALP ≥1.67 × ULN based on studies with a single baseline biochemistry measurement [12,13]. A growing body of literature suggests a continued risk of negative outcomes (disease progression, liver transplantation and death) even in patients meeting the various ALP criteria used to define biochemical treatment response, while also highlighting the potential predictive value of total bilirubin (TB), AST, alanine transaminase (ALT) and serum albumin [14–19]. While UDCA nonresponse and key predictors of negative clinical outcomes are defined biochemically, liver stiffness measurements offer complementary information that may indicate ongoing disease activity or incomplete disease control, even when biochemical response appears adequate [20].

Several risk prediction studies have extrapolated outcomes based on limited biomarker data (i.e., at baseline and after a short duration of first-line treatment, or by evaluating only a small set of biomarkers). The value of single-point-in-time or short-term biomarker levels is limited by multiple factors (e.g., ALP can be elevated following a bone fracture). Furthermore, biomarker levels at baseline do not reflect the effectiveness of long-term disease management over years to decades of follow-up. The association between clinical outcomes and a biomarker measurement outside the threshold may have a cumulative effect over time in this chronic disease. It is conceivable that persistent values beyond thresholds for multiple biochemical markers may be associated with a greater risk than a single occurrence of increased or decreased levels of a single biomarker.

The aim of this study is to expand on previous work examining the associations of longitudinal measurements of several liver biomarkers in risk assessment modeling in PBC, using real-world data from a large administrative claims database [16,17]. We analyzed repeated liver biochemistry measurements to explore the association between the degree and duration of time outside of normal limits for ALP, TB, AST, ALT and albumin with negative clinical outcomes, defined as a composite outcome of time to first occurrence of hospitalization for hepatic decompensation, liver transplantation or death among patients with PBC.

## Materials & methods

### Data source

This retrospective cohort study was conducted using Optum's de-identified Clinformatics^®^ Data Mart Database (CDM), which is derived from a database of administrative health claims for members of large commercial and Medicare Advantage health plans. CDM also includes laboratory test results.

### Study population

Patients aged ≥18 years, without cirrhosis or with compensated cirrhosis, and enrolled in the CDM database from 1 July 2007 to 31 March 2021, were included if they were diagnosed with PBC (index date) by either ≥2 outpatient claims with a PBC diagnosis (ICD-10-CM K74.3 or ICD-9-CM 571.6) on separate days or ≥1 inpatient claim with a diagnosis of PBC; had ≥6 months of continuous enrollment prior to PBC diagnosis, including the index date (baseline period); and had lab values during the baseline period or up to 14 days post index and ≥1 laboratory value in the post index period.

Patients were excluded if they had confounding comorbidities including autoimmune hepatitis (AIH), HIV infection, history of gastric bypass, any history of hepatitis C or active hepatitis B infection, primary sclerosing cholangitis, alcohol-associated liver disease, Gilbert syndrome, hepatocellular carcinoma, hepatorenal syndrome, Paget’s disease, recent bone fracture, portal hypertension or evidence of hepatic decompensation (diagnosis, procedure and drug codes in Supplementary Table 1). Patients were also excluded if they had received a liver transplant any time prior to index or had abnormally high baseline liver biochemistry values (ALP >1000 u/l, TB >3 mg/dl, AST >300 u/l, or ALT >300 u/l) in the 6 months prior to index. Patients who received any second-line therapies, either obeticholic acid (OCA) or off-label fenofibrate, at baseline were excluded to eliminate this as a potential confounder.

### Time-dependent exposure variable

The primary analysis was a survival analysis using a Cox model with a time-dependent exposure variable, whereby predictor variables were measured repeatedly throughout the study period to estimate cumulative effects over time. We quantified exposure as the proportion of time a biomarker level exceeded a prespecified threshold [21]. For example, using a threshold for TB of ULN, a patient with TB > ULN for 12 months over 2 years would have a value beyond normal limits 50% of the time by the end of 2 years; if there were no further TB elevations, the patient would be elevated 33% of the time at 3 years and 20% of the time at 5 years. Thus, the percentage of time TB is elevated changes as a function of time under observation. The hazard ratio (HR) for an increase in the proportion of time outside of normal limits was expressed in 10% increments. Laboratory values were carried forward between observations.

The thresholds chosen for this analysis capture those used in common UDCA response criteria (e.g., Paris I, Paris II and Rotterdam) [8,22]. The thresholds for each biomarker were defined as multiples of ULN for ALP (1×, 1.2×, 1.5×, 1.67×, 2.0×), TB (0.6×, 0.8×, 1.0×, 2.0×), AST (1×, 1.5×, 2.0×) and ALT (1×, 1.5×, 2.0×) and multiples of the lower limits of normal (LLN) for albumin (1×, 1.09×, 1.14×). For TB, recent research has shown that levels above 0.6 × ULN are associated with incremental risk of liver transplantation and death in patients with PBC [18], so thresholds were examined at and above this level.

### Baseline predictor variables

Baseline variables in the models included patient demographics (age and sex) and comorbidities (nonalcoholic steatohepatitis [NASH] and cirrhosis). Baseline comorbidities were coded as present if diagnosis or procedure codes were present in the 6 months before index (diagnosis, procedure, and drug codes in Supplementary Table 1).

### Outcome variables

Negative clinical outcomes were defined as a composite of first occurrence of hospitalization for a decompensating event, liver transplantation, or all-cause death outcomes commonly utilized in PBC studies [23]. Decompensating events included ascites, esophageal or gastric varices with bleeding, or hepatic encephalopathy. The use of spironolactone, furosemide, lactulose, or rifaximin during an inpatient admission was also considered indicative of hepatic decompensation.

### Statistical analysis

Baseline patient characteristics were reported using descriptive statistics. Frequency and timing of liver biochemistry testing during the follow-up period were also characterized. The number and percentage of subjects either censored or with outcomes were presented.

The primary modeling analyses employed a time-dependent Cox proportional hazards model with time-dependent covariates in survival analyses, assessing time to first occurrence hospitalization for hepatic decompensation, liver transplantation, or death. Time-invariant baseline covariates included age, sex, NASH and cirrhosis. The time-dependent exposure was the proportion of laboratory values outside prespecified thresholds at each time point (summary of thresholds in Table 2). Patients were censored at database disenrollment, initiation of either OCA or fenofibrate or the end of the study period.

**Table 1.** Baseline and follow-up patient characteristics and negative clinical outcome rates.

Baseline characteristic	Frequency/mean (n = 2402)
Sex, n (%)	
Female	2049 (85.3)
Male	353 (14.7)
Age, mean (SD), y	63.3 (13.1)
NASH, n (%)	169 (7.0)
Cirrhosis, n (%)	265 (11.0)
Baseline UDCA, n (%)	1637 (68.2)
UDCA use during follow-up, n (%)	1697 (70.7)
**Follow-up characteristic**	
Follow-up time, median (IQR), y	2.2 (1.1–3.9)
Censoring events, n (%)	
Follow-up OCA or fenofibrate	87 (3.6)
End of enrollment	1729 (72.0)
End of follow-up	395 (16.4)
Outcomes, n (%)	
Composite	191 (8.0)
First event^†^	
Hospitalization for hepatic decompensation	99 (4.1)
Liver transplant	5 (0.2)
Death	87 (3.6)

† Events are not mutually exclusive; multiple events can occur on the same date.

IQR: Interquartile range; NASH: Nonalcoholic steatohepatitis; OCA: Obeticholic acid; SD: Standard deviation; UDCA: Ursodeoxycholic acid.

**Table 2.** Follow-up patient characteristics and negative clinical outcome rates in each model.

Characteristic	Frequency/mean
ALP model (n = 2378)	
Follow-up time, median (IQR)	2.2 (1.1–3.9)
Follow-up ALP labs, n, mean (SD)	5.4 (5.2)
Patients with follow-up labs outside of specified threshold, n (%)	
ALP ≥ULN	1635 (68.8)
ALP ≥1.2 × ULN	1305 (54.9)
ALP ≥1.5 × ULN	933 (39.2)
ALP ≥1.67 × ULN	777 (32.7)
ALP ≥2 × ULN	550 (23.1)
Proportion/percentage of follow-up time outside of normal limits, mean (SD)	
ALP ≥ULN	52.5 (44.6)
ALP ≥1.2 × ULN	38.3 (43.6)
ALP ≥1.5 × ULN	23.9 (37.8)
ALP ≥1.67 × ULN	18.4 (34.2)
ALP ≥2 × ULN	12.3 (28.9)
Censoring events, n (%)	
Follow-up OCA or fenofibrate	86 (3.6)
End of enrollment	1710 (72.0)
End of follow-up	394 (16.6)
Outcomes, n (%)	
Composite	188 (7.9)
First event^†^	
Hospitalization for hepatic decompensation	98 (4.1)
Liver transplant	5 (0.2)
Death	85 (3.6)
**TB model (n = 2373)**	
Follow-up time, median (IQR)	2.2 (1.1–3.9)
Follow-up TB labs, n, mean (SD)	5.4 (5.2)
Patients with follow-up labs outside of specified threshold, n (%)	
TB ≥0.6 × ULN	1264 (53.3)
TB ≥0.8 × ULN	675 (28.5)
TB ≥ULN	425 (17.9)
TB ≥2 × ULN	87 (3.7)
Proportion/percentage of follow-up time outside of normal limits, mean (SD)	
TB ≥0.6 × ULN	31.5 (39.3)
TB ≥0.8 × ULN	15.3 (30.9)
TB ≥ULN	8.5 (23.6)
TB ≥2 × ULN	1.1 (7.9)
Censoring events, n (%)	
Follow-up OCA or fenofibrate	86 (3.6)
End of enrollment	1708 (72.0)
End of follow-up	391 (16.5)
Outcomes, n (%)	
Composite	188 (7.9)
First event^†^	
Hospitalization for hepatic decompensation	98 (4.1)
Liver transplant	5 (0.2)
Death	85 (3.6)
**AST model (n = 2378)**	
Follow-up time, median (IQR)	2.2 (1.1–3.9)
Follow-up AST labs, n, mean (SD)	5.4 (5.2)
Patients with follow-up labs outside of specified threshold, n (%)	
AST ≥ULN	1489 (62.6)
AST ≥1.5 × ULN	784 (33.0)
AST ≥2 × ULN	478 (20.1)
AST ≥5 × ULN	49 (2.1)
Proportion/percentage of follow-up time outside of normal limits, mean (SD)	
AST ≥ULN	37.5 (40.9)
AST ≥1.5 × ULN	14.6 (29.3)
AST ≥2 × ULN	7.5 (21.5)
AST ≥5 × ULN	0.3 (2.8)
Censoring events, n (%)	
Follow-up OCA or fenofibrate	86 (3.6)
End of enrollment	1711 (72.0)
End of follow-up	393 (16.5)
Outcomes, n (%)	
Composite	188 (7.9)
First event^†^	
Hospitalization for hepatic decompensation	98 (4.1)
Liver transplant	5 (0.2)
Death	85 (3.6)
**ALT model (n = 2385)**	
Follow-up time, median (IQR)	2.2 (1.1–3.9)
Follow-up ALT labs, n, mean (SD)	5.4 (5.2)
Patients with follow-up labs outside of specified threshold, n (%)	
ALT ≥ULN	1020 (42.8)
ALT ≥1.5 × ULN	555 (23.3)
ALT ≥2 × ULN	337 (14.1)
ALT ≥5 × ULN	29 (1.2)
Proportion/percentage of follow-up time outside of normal limits, mean (SD)	
ALT ≥ULN	20.7 (33.5)
ALT ≥1.5 × ULN	9.0 (23.0)
ALT ≥2 × ULN	4.8 (17.1)
ALT ≥5 × ULN	0.2 (3.6)
Censoring events, n (%)	
Follow-up OCA or fenofibrate	86 (3.6)
End of enrollment	1717 (72.0)
End of follow-up	392 (16.4)
Outcomes, n (%)	
Composite	190 (8.0)
First event^†^	
Hospitalization for hepatic decompensation	99 (4.2)
Liver transplant	5 (0.2)
Death	86 (3.6)
**Albumin model (n = 2383)**	
Follow-up time, median (IQR)	2.2 (1.1–3.9)
Follow-up ALB labs, n, mean (SD)	5.5 (5.3)
Patients with follow-up labs outside of specified threshold, n (%)	
ALB ≤1.14 × LLN	1149 (48.2)
ALB ≤1.09 × LLN	926 (38.9)
ALB ≤1.0 × LLN	436 (18.3)
Proportion/percentage of follow-up time outside of normal limits, mean (SD)	
ALB ≤1.14 × LLN	26.3 (36.7)
ALB ≤1.09 × LLN	19.2 (32.7)
ALB ≤1.0 × LLN	7.2 (21.2)
Censoring events, n (%)	
Follow-up OCA or fenofibrate	86 (3.6)
End of enrollment	1716 (72.0)
End of follow-up	391 (16.4)
Outcomes, n (%)	
Composite	190 (8.0)
First event^†^	
Hospitalization for hepatic decompensation	98 (4.1)
Liver transplant	5 (0.2)
Death	87 (3.7)

† Events are not mutually exclusive; multiple events can occur on the same date.

Follow-up time, censoring events, negative clinical outcomes, and proportion of follow-up time with laboratory values outside of evaluated thresholds are shown for each model.

ALB: Albumin; ALP: Alkaline phosphatase; ALT: Alanine transaminase; AST: Aspartate aminotransferase; IQR: Interquartile range; OCA: Obeticholic acid; SD: Standard deviation; TB: Total bilirubin; ULN: Upper limit of normal.

Separate models were estimated for the proportion of time spent above thresholds for each biomarker. Another model including both ALP and TB, choosing the lowest threshold for each variable (ALP ≥ULN and TB ≥ 0.6 × ULN) was also estimated. The proportional hazards assumption was tested using a global chi-square test based on the scaled Schoenfeld residuals. HRs and 95% CIs for each covariate and proportion of time above thresholds (per 10% increment) were reported.

Two subgroup sensitivity analyses using Cox proportional models were performed. The first sensitivity analysis included only patients with a PBC diagnosis identified based on ICD-10 codes, and the second sensitivity analysis excluded patients with NASH or cirrhosis at baseline. HRs, lower and upper confidence levels (CLs), and p*-*values were reported.

All analyses were conducted in SAS v9.4 and R v4.0.0.

## Results

### Patient characteristics

**Figure 1.**
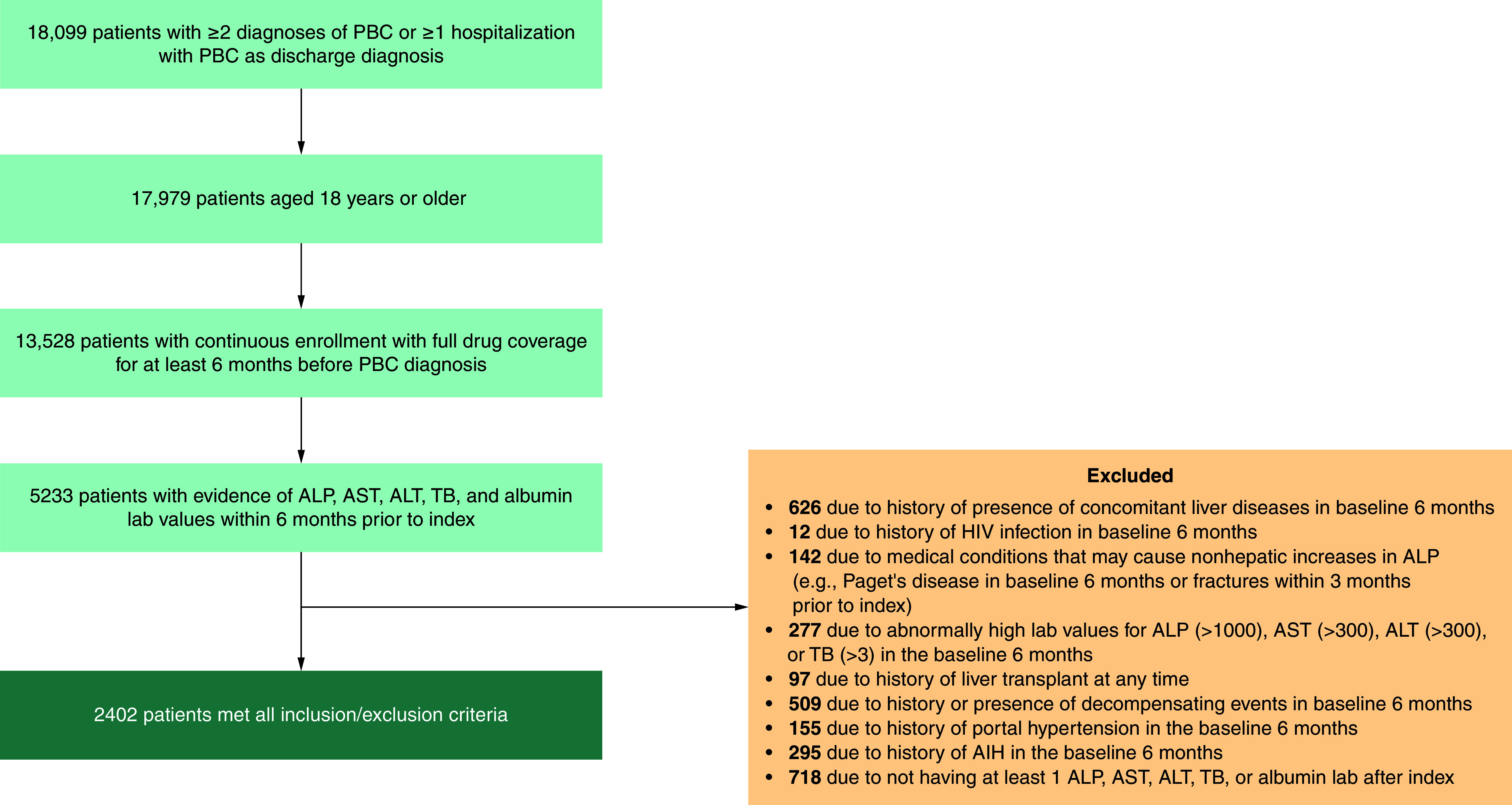
Flow diagram of patient selection. AIH: Autoimmune hepatitis; ALP: Alkaline phosphatase; ALT: Alanine aminotransferase; AST: Aspartate aminotransferase; PBC: Primary biliary cholangitis; TB: Total bilirubin.

Of 17,979 adult patients in the database with a diagnosis of PBC, 2402 fulfilled inclusion criteria (Figure 1). As shown in Table 1, 85.3% of patients were female. At baseline, mean patient age was 63.3 years (SD: 13.1); 11.0% had compensated cirrhosis, 7.0% had NASH and 68.2% were receiving UDCA therapy. During follow-up, 70.7% of patients received UDCA. Median follow-up time was 2.2 years (interquartile range: 1.1–3.9 years); 3.6% (n = 87) of patients were censored at the start of second-line treatment. Overall, 8.0% (n = 191) experienced ≥1 negative clinical outcome; the first event was hospitalization for hepatic decompensation in 4.1% of patients (n = 99), liver transplantation in 0.2% (n = 5), and death in 3.6% (n = 87) (Table 1).

Among the 2402 patients identified, a total of 2378 patients met the criteria and were included in the ALP model; 2373 were included in the TB model, 2378 were included in the AST model, 2385 were included in the ALT model, and 2383 were included in the albumin model. The combined ALP and TB model included 2370 patients. Follow-up time, censoring, negative clinical outcomes and proportions of time outside of evaluated thresholds are presented in Table 2. On average, patients had approximately five measures of each biomarker prior to censoring or an event, whichever occurred first. Mean proportion of time above ULN was 52.5% for ALP, 8.5% for TB, 37.5% for AST and 20.7% for ALT; mean proportion of time below LLN was 7.2% for albumin.

### Negative clinical outcomes

**Figure 2.**
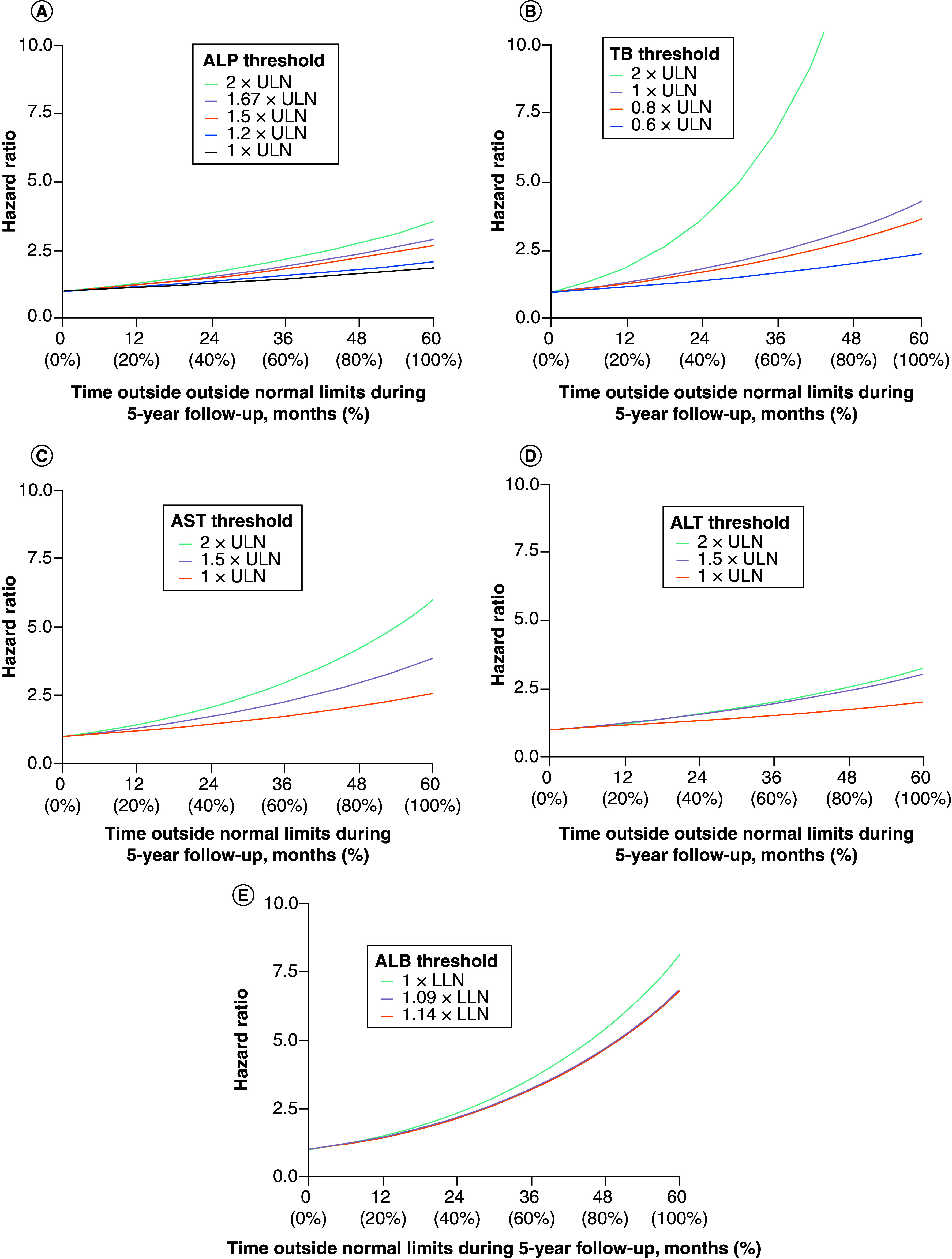
Hazard ratios for risk of hospitalization for hepatic decompensation, liver transplantation or death (composite) as a function of time above prespecified thresholds. 5-year follow-up example for: **(A)** ALP (n = 2379), **(B)** total bilirubin (n = 2374), **(C)** AST (n = 2379), **(D)** ALT (n = 2386) and **(E)** ALB (n = 2384). ALB: Albumin; ALP: Alkaline phosphatase; ALT: Alanine aminotransferase; AST: Aspartate aminotransferase; LLN: Lower limit of normal; TB: Total bilirubin; ULN: Upper limit of normal.

For each of the biomarkers assessed (ALP, TB, AST, ALT and albumin), a greater proportion of time spent outside of thresholds and greater divergence from normal limits were associated with an increased risk of hepatic decompensation, liver transplantation, or death (Figure 2). Using the 5-year modeled follow-up period as an example, a patient with TB > ULN for 12 months (20% of 5 years) was estimated to have a 34% increase in risk (HR = 1.34) of negative clinical outcomes (Figure 2B), while a patient with 36 months of TB > ULN over 5 years (60% of the time) would have a 140% (2.4-times greater) risk (HR = 2.40). For ALP (Figure 2A), AST (Figure 2C) and ALT (Figure 2D), each 10% increase in time above ULN was associated with a 6.5%, 9.9% and 7.3% increase in risk, respectively. For albumin, each 10% increase in time below LLN was associated with a 23.3% increase in risk (Figure 2E).

The assumptions of proportional hazard, evaluated using a global chi-square test based on the scaled Schoenfeld residuals for each model, were supported by nonsignificant global tests for all thresholds with the exceptions of ALP ≥ 2 × ULN (p = 0.007) and TB ≥ 2 × ULN (p = 0.006).

**Figure 3.**
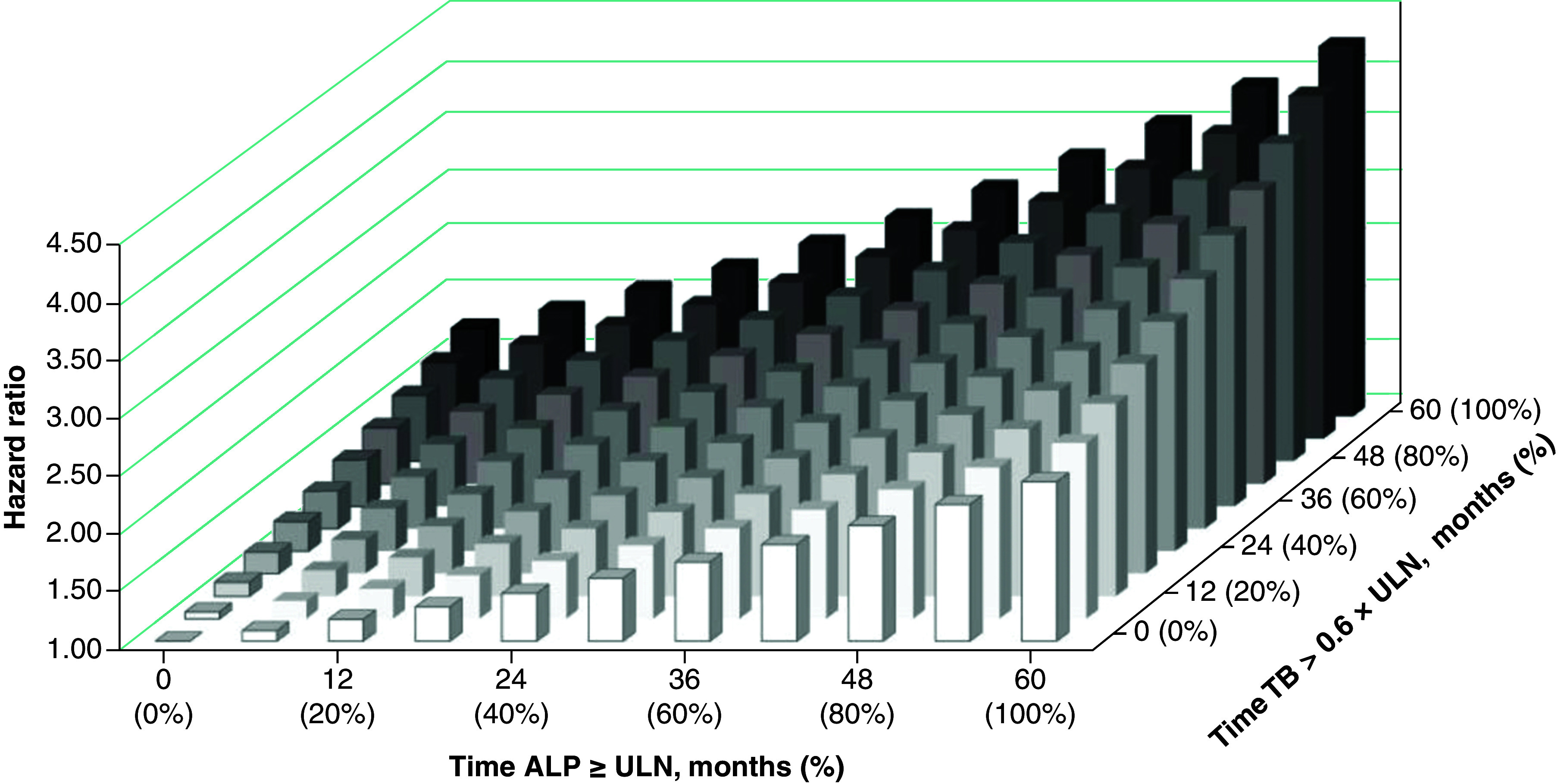
Hazard ratios for risk of hospitalization for hepatic decompensation, liver transplantation or death (composite) as a function of time above prespecified thresholds. Five-year follow-up example for ALP ≥ULN and total bilirubin ≥ 0.6 × ULN (n = 2370). ALP: Alkaline phosphatase; TB: Total bilirubin; ULN: Upper limit of normal.

Table 3 shows the Cox regression model with time-dependent covariates combining ALP and TB, with the lowest thresholds applied. All variables except NASH were associated with a significant increase in risk of a negative clinical outcome. As shown in Figure 3, there was a fourfold increase in the risk of an event in patients with consistent elevations (100% of observed time) of both ALP ≥ULN and TB ≥ 0.6 × ULN relative to patients with no time above these thresholds. Supplementary Table 2 demonstrates the significant increase in risk of a negative clinical outcome as ALP and TB deviate further from ULN.

**Table 3.** Hazard ratios for risk of hospitalization for hepatic decompensation, liver transplant or death (composite) as a function of time above prespecified thresholds from Cox proportional hazards model with time-dependent exposure.

Parameter	Hazard ratio	95% CI	p-value
Proportion of time ALP ≥ ULN (for a 10% increase)	1.059	1.033–1.085	<0.001
Proportion of time TB ≥ 0.6 × ULN (for a 10% increase)	1.091	1.063–1.119	<0.001
Age (per 10-year increase)	1.568	1.415–1.737	<0.001
Sex (female vs male)	0.609	0.472–0.785	<0.001
NASH (yes vs no)	1.385	0.967–1.984	0.076
Cirrhosis (yes vs no)	1.470	1.098–1.68	0.010

n = 2370.

The primary analysis was a survival analysis using a Cox proportional hazards model with a time-dependent exposure variable. Predictor variables were measured repeatedly throughout the study period to estimate cumulative effects over time. The effects of age, sex, and the presence of NASH or cirrhosis were also evaluated.

ALP: Alkaline phosphatase; CI: Confidence interval; NASH: Nonalcoholic steatohepatitis; TB: Total bilirubin; ULN: Upper limit of normal.

### Subgroup sensitivity analyses

Results from a sensitivity analysis (Supplementary Tables 3 & 4) including only patients with a confirmed PBC diagnosis based on ICD-10 codes (n = 1532) and a separate sensitivity analysis (Supplementary Tables 5 & 6) excluding patients with NASH or cirrhosis at baseline (n = 1977) were similar to results from the original analyses.

## Discussion

In this study, 8.0% of patients with PBC, either without cirrhosis or with compensated cirrhosis, experienced negative clinical outcomes, defined as a composite of the first occurrence of hospitalization for hepatic decompensation, liver transplantation or death. The most common first event of the composite outcome was hospitalization for hepatic decompensation (4.1%), indicative of meaningful disease progression during follow-up. Death was the first event for 3.6% of patients; the mortality component of the composite end point, defined as all-cause death, aligns with common reporting in PBC studies [23]. Liver transplantation was the least frequent first event (0.2%). ALP, TB, AST, ALT and albumin values beyond thresholds were each associated with an increased risk of negative clinical outcomes. Additionally, in line with previous reports [18], the risk of the negative clinical outcome was fourfold higher in patients with both ALP ≥ ULN and TB ≥ 0.6 × ULN during the follow-up period.

Current AASLD and European Association for the Study of the Liver guidelines are consistent with the seminal works of Lammers *et al.* [16] and Carbone *et al.* [17] in utilizing single measures of biomarkers to predict the risk of liver transplantation and death, whereby ALP values > 1.67 × ULN and >1.5 × ULN and TB > ULN were identified as thresholds triggering the need for second-line pharmaceutical intervention in PBC [12,13]. Although the GLOBE [24] and UK-PBC [17] scores are designed to predict transplant-free and liver-related outcomes, respectively, they are based on static measurements taken at baseline and 12 months after initiating UDCA treatment, rather than continuous monitoring over the treatment period. The findings of the current analysis build on this existing evidence, highlighting the clinical relevance of AST, ALT and albumin levels, while continuing to underscore the significance of elevated ALP and TB levels. Furthermore, these results emphasize the importance of continued biomarker monitoring throughout the disease course, given the observed increases in the composite outcome risk associated with not only the magnitude of out-of-range biomarker values but also with the duration of time spent outside the thresholds.

These findings suggest that clinicians and treatment guidelines should expand beyond monitoring only ALP and TB when assessing risk or determining the need to initiate first- or second-line therapy, by incorporating additional biomarkers such as ALT, AST and albumin. This work may support the evolution of biochemical criteria used in PBC disease monitoring. The importance of timely pharmacotherapeutic intervention in PBC is highlighted by the reduction in negative clinical outcomes demonstrated with first-line UDCA and second-line OCA treatment [25–32]. Additionally, PBC management may benefit from considering both the absolute levels of multiple biomarkers and the duration of time spent beyond thresholds when evaluating risk of disease progression and guiding treatment decisions for the appropriate patient populations. Future research is needed to expand on these findings to further validate and quantify the potential benefits of timely therapeutic intervention in PBC, including examining the cumulative effect of threshold deviations across multiple biomarkers and measures of inflammation and fibrosis.

### Limitations

This was a retrospective analysis of administrative claims data, which has inherent limitations, including the possibility of miscoding, billing-related bias and missing information. To mitigate the risk of miscoding, we required at least two outpatient or one inpatient claims with a PBC diagnosis, as similar criterion has demonstrated a positive predictive value of 73% for confirmed PBC and 89% for confirmed or suspected PBC [33]. Prior to October 2015, when no PBC-specific ICD-9 diagnosis code existed, identification was based on the biliary cirrhosis code (571.6). However, our sensitivity analysis, which included only patients with an ICD-10 PBC-specific diagnosis code, confirmed the original study findings. Additionally, although we excluded patients with an AIH diagnosis code at baseline, it is possible that a small percentage of patients with a PBC/AIH overlap syndrome were included.

Patients receiving second-line treatments were excluded from this analysis in order to isolate outcomes under standard first-line therapy (i.e., UDCA). As a result, the study cohort may underrepresent individuals at higher risk for negative clinical outcomes. Although liver stiffness can also be used to show inadequate response to UDCA and progression of disease [13,20], liver stiffness measurements data are not readily available in claims data.

It should be noted that patients who met the study inclusion criteria entered the cohort at the time of their first qualifying PBC diagnosis during the prespecified study period. As a result, the patient population included both incident and prevalent PBC, likely with heterogeneous time since their initial diagnosis. Additionally, patients had varying lengths of follow-up due to changes in their insurance status. Our study also included patients with NASH and cirrhosis, conditions that, when combined with PBC, may lead to negative clinical outcome sooner than with PBC alone. However, a separate sensitivity analysis excluding individuals with NASH or cirrhosis at baseline demonstrated that our findings remained qualitatively unchanged. The significance and magnitude of the effect of increasing time outside biomarker thresholds in the subgroup were similar to those observed in the overall analyses.

Regarding the composite outcome, cause of death was not reported in this study, as this information is not readily available in claims data; however, patients with PBC often have a high comorbidity burden, which may have contributed to mortality [34]. Additionally, the biomarkers and thresholds of interest (ALP, AST, and ALT >ULN, TB >0.6 × ULN, and albumin <LLN) were based on laboratory test results. Extreme/implausible values were removed to avoid associations driven by outliers, and patients with no laboratory tests during the follow-up period were excluded, potentially introducing selection and measurement bias. Unlike clinical trial data, laboratory assessments in clinical practice are not collected at fixed intervals, and not all lab values were available in the dataset. As a result, laboratory values were carried forward between observations and may not capture potential changes that occurred during these intervals. Additionally, adjustments were not made for other key prognostic factors, such as UDCA response, fibrosis stage, comorbidities, sex or treatment adherence, potentially introducing residual confounding. Finally, as with any real-world study, unobserved factors and limitations in generalizability are potential concerns, as this study focused on patients with compensated PBC who had US insurance coverage. However, the use of a large US healthcare database, along with prespecified inclusion and exclusion criteria and replication of results across multiple sensitivity analyses, supports the robustness of the findings.

In conclusion, this research demonstrates that in patients living with PBC, whether untreated or receiving first-line therapy, elevations in ALP, TB, AST and ALT and reduction in albumin levels beyond guideline-recommended thresholds are predictive of negative clinical outcomes. A combination of ALP and TB above conservative thresholds was a robust predictor, while the aminotransferases AST and ALT yielded additional insight into the risk of negative outcomes. Our findings further suggest that longitudinal biomarker monitoring may offer additional value by enabling earlier risk stratification and identification of UDCA nonresponse prior to the 12-month time point used in existing prognostic scores and biochemical response criteria. Given evidence demonstrating the ability of current therapies to reduce these events [31,35–37], clinicians, and ultimately, treatment guidelines may consider more frequent monitoring of all available liver biomarkers and timely initiation of treatment to reduce the risk of negative clinical outcomes.

### Summary points

- Primary biliary cholangitis (PBC) is a rare chronic liver disease that, without effective treatment, may progress to cirrhosis and lead to negative clinical outcomes, including hepatic decompensation, liver transplantation, and death.
- Current treatment guidelines recommend monitoring alkaline phosphatase (ALP) and total bilirubin (TB) to determine treatment response and predict the risk of negative clinical outcomes.
- This retrospective cohort study used administrative health claims and laboratory test results from patients with PBC enrolled in Optum's de-identified Clinformatics^®^ Data Mart Database to analyze repeated liver biochemistry measurements.
- A time-dependent Cox proportional hazards model with time-dependent covariates assessed the time to first occurrence of hepatic decompensation, liver transplantation, or death.
- Separate models evaluated the association between the degree and duration of time outside normal limits for ALP, TB, alanine aminotransferase (ALT), aspartate aminotransferase (AST) and albumin with negative clinical outcomes.
- Between 2007 and 2021, 2402 patients met the inclusion criteria. Among these patients, 8.0% experienced a negative clinical outcome of hepatic decompensation, liver transplantation or death.
- Each 10% increase in time outside thresholds was associated with a 6.5%, 9.2%, 9.9%, 7.3% and 23.3% increase in risk of negative outcomes for ALP, TB, AST, ALT and albumin, respectively.
- For each biomarker, a greater proportion of time spent outside recommended thresholds and a greater divergence from normal limits correlated with an increased risk of negative clinical outcomes, suggesting that more frequent monitoring of all available liver biomarkers and timely treatment initiation may improve patient outcomes.

## Supplementary Material


