# Supplementary material for: Association of liver biomarker values beyond current thresholds and negative clinical outcomes in primary biliary cholangitis: analysis of a real-world healthcare claims database
Source: J Comp Eff Res. 2025 Nov 18;14(12):e240198. doi: 10.57264/cer-2024-0198 (PMC12679663; doi:10.57264/cer-2024-0198)
Supplement: Supplementary file 1 [file cer-14-240198-s1.docx]

**Appendix:** Supplementary Material

**Contents**

[**SUPPLEMENTARY TABLES** 2](#_Toc182490793)

[**Supplemental Table 1.** Diagnosis, Procedure, and Drug Codes 2](#_Toc182490794)

[**Supplemental Table 2.** Original Cox Proportional Model With Individual Uncontrolled ALP and Uncontrolled Bilirubin (N=2370) 36](#_Toc182490795)

[**Supplemental Table 3.** Sensitivity Analysis #1 (Cox Proportional Model Among Only Patients With a Confirmed PBC Diagnosis Based on ICD-10 Codes [N=1532]) 37](#_Toc182490796)

[**Supplemental Table 4.** Sensitivity Analysis #1 (Cox Proportional Model With Individual Uncontrolled ALP and Uncontrolled Bilirubin Among Only Patients With a Confirmed PBC Diagnosis Based on ICD-10 Codes [N=1532]) 38](#_Toc182490797)

[**Supplemental Table 5.** Sensitivity Analysis #2 (Cox Proportional Model Among Patients Without NASH or Cirrhosis [N=1977]) 40](#_Toc182490798)

[**Supplemental Table 6.** Sensitivity Analysis #2 (Cox Proportional Model With Individual Uncontrolled ALP and Uncontrolled Bilirubin Among Patients Without NASH or Cirrhosis [N=1977]) 41](#_Toc182490799)

**SUPPLEMENTARY TABLES**

**Supplemental Table 1.** Diagnosis, Procedure, and Drug Codes

| **Condition/Procedure/Treatment** | **Code Type** | **Code** | **Description** |
| --- | --- | --- | --- |
| **Hepatitis C virus infection** | ICD-10 | B17.10 | Acute hepatitis C without hepatic coma |
|  | ICD-10 | B17.11 | Acute hepatitis C with hepatic coma |
|  | ICD-9 | 070.41 | Acute hepatitis C with hepatic coma |
|  | ICD-9 | 070.51 | Acute hepatitis C without mention of hepatic coma |
|  | ICD-9 | 070.70 | Unspecified viral hepatitis C without hepatic coma |
|  | ICD-9 | 070.71 | Unspecified viral hepatitis C with hepatic coma |
| **Active hepatitis B virus infection** | ICD-10 | B16.0 | Acute hepatitis B with delta-agent with hepatic coma |
|  | ICD-10 | B16.1 | Acute hepatitis B with delta-agent without hepatic coma |
|  | ICD-10 | B16.2 | Acute hepatitis B without delta-agent with hepatic coma |
|  | ICD-10 | B16.9 | Acute hepatitis B without delta-agent and without hepatic coma |
|  | ICD-10 | B17.0 | Acute delta-(super) infection of hepatitis B carrier |
|  | ICD-9 | 070.20 | Viral hepatitis B with hepatic coma, acute or unspecified, without mention of hepatitis delta |
|  | ICD-9 | 070.21 | Viral hepatitis B with hepatic coma, acute or unspecified, with hepatitis delta |
|  | ICD-9 | 070.30 | Viral hepatitis B without mention of hepatic coma, acute or unspecified, without mention of hepatitis delta |
|  | ICD-9 | 070.31 | Viral hepatitis B without mention of hepatic coma, acute or unspecified, with hepatitis delta |
| **Primary sclerosing cholangitis** | ICD-10 | K83.01 | Primary sclerosing cholangitis (2019-2021 only) |
| **Alcoholic liver disease** | ICD-10 | K70.0 | Alcoholic fatty liver |
|  | ICD-10 | K70.10 | Alcoholic hepatitis without ascites |
|  | ICD-10 | K70.11 | Alcoholic hepatitis with ascites |
|  | ICD-10 | K70.2 | Alcoholic fibrosis and sclerosis of liver |
|  | ICD-10 | K70.30 | Alcoholic cirrhosis of liver without ascites |
|  | ICD-10 | K70.31 | Alcoholic cirrhosis of liver with ascites |
|  | ICD-10 | K70.40 | Alcoholic hepatic failure without coma |
|  | ICD-10 | K70.41 | Alcoholic hepatic failure with coma |
|  | ICD-10 | K70.9 | Alcoholic liver disease, unspecified |
|  | ICD-9 | 571.0 | Alcoholic fatty liver |
|  | ICD-9 | 571.1 | Acute alcoholic hepatitis |
|  | ICD-9 | 571.2 | Alcoholic cirrhosis of liver |
|  | ICD-9 | 571.3 | Alcoholic liver damage, unspecified |
| **Autoimmune liver disease or autoimmune hepatitis** | ICD-10 | K75.4 | Autoimmune hepatitis |
|  | ICD-9 | 571.42 | Autoimmune hepatitis |
| **Nonalcoholic steatohepatitis** | ICD-10 | K75.81 | Nonalcoholic steatohepatitis |
|  | ICD-9 | 571.8 | Other chronic nonalcoholic liver disease |
| **Gilbert syndrome** | ICD-10 | E80.4 | Gilbert syndrome |
|  | ICD-9 | 277.4 | Disorders of bilirubin excretion |
| **Esophageal varices with bleeding** | ICD-10 | I85.01 | Esophageal varices with bleeding |
|  | ICD-10 | I85.11 | Secondary esophageal varices with bleeding |
|  | ICD-9 | 456.1 | Esophageal varices without mention of bleeding |
|  | ICD-9 | 456.21 | Esophageal varices in diseases classified elsewhere, without mention of bleeding |
| **Ascites** | ICD-10 | K70.11 | Alcoholic hepatitis with ascites |
|  | ICD-10 | K70.31 | Alcoholic cirrhosis of liver with ascites |
|  | ICD-10 | K71.51 | Fluid in peritoneal cavity |
|  | ICD-10 | R18.0 | Malignant ascites |
|  | ICD-10 | R18.8 | Other ascites |
|  | ICD-9 | 567.23 | Spontaneous bacterial peritonitis |
|  | ICD-9 | 571.2 | Alcoholic cirrhosis of liver |
|  | ICD-9 | 789.51 | Malignant ascites |
|  | ICD-9 | 789.59 | Other ascites |
| **Hepatic encephalopathy** | ICD-10 | B15.0 | Hepatitis A with hepatic coma |
|  | ICD-10 | B16.0 | Acute hepatitis B with delta-agent with hepatic coma |
|  | ICD-10 | B16.2 | Acute hepatitis B without delta-agent with hepatic coma |
|  | ICD-10 | B17.11 | Acute hepatitis C with hepatic coma |
|  | ICD-10 | B19.0 | Unspecified viral hepatitis with hepatic coma |
|  | ICD-10 | B19.11 | Unspecified viral hepatitis B with hepatic coma |
|  | ICD-10 | B19.21 | Unspecified viral hepatitis C with hepatic coma |
|  | ICD-10 | K70.41 | Alcoholic hepatic failure with coma |
|  | ICD-10 | K72.11 | Chronic hepatic failure with coma |
|  | ICD-10 | K72.90 | Hepatic failure, unspecified without coma |
|  | ICD-10 | K72.91 | Hepatic failure, unspecified with coma |
|  | ICD-9 | 572.2 | Hepatic encephalopathy |
|  | ICD-9 | 070.0 | Viral hepatitis A with hepatic coma |
|  | ICD-9 | 070.21 | Viral hepatitis B with hepatic coma, acute or unspecified, with hepatitis delta |
|  | ICD-9 | 070.20 | Viral hepatitis B with hepatic coma, acute or unspecified, without mention of hepatitis delta |
|  | ICD-9 | 070.41 | Acute hepatitis C with hepatic coma |
|  | ICD-9 | 070.6 | Unspecified viral hepatitis with hepatic coma |
|  | ICD-9 | 070.71 | Unspecified viral hepatitis C with hepatic coma |
| **Gastric varices** | ICD-10 | I86.4 | Gastric varices |
|  | ICD-9 | 456.8 | Varices of other sites (nonspecific) |
| **Esophageal varices without bleeding** | ICD-10 | I85.00 | Esophageal varices without bleeding |
|  | ICD-10 | I85.10 | Secondary esophageal varices without bleeding |
|  | ICD-9 | 456.1 | Esophageal varices without mention of bleeding |
|  | ICD-9 | 456.21 | Esophageal varices in diseases classified elsewhere, without mention of bleeding |
| **Portal hypertension** | ICD-10 | K76.6 | Portal hypertension |
|  | ICD-9 | 572.3 | Portal hypertension |
| **Hepatocellular carcinoma** | ICD-10 | C22.0 | Liver cell carcinoma |
|  | ICD-10 | C22.8 | Malignant neoplasm of liver, primary, unspecified as to type |
|  | ICD-10 | C22.9 | Malignant neoplasm of liver, not specified as primary or secondary |
|  | ICD-9 | 155.0 | Malignant neoplasm of liver, primary |
|  | ICD-9 | 155.2 | Malignant neoplasm of liver, not specified as primary or secondary |
| **Hepatorenal syndrome** | ICD-10 | K76.7 | Hepatorenal syndrome |
|  | ICD-9 | 572.4 | Hepatorenal syndrome |
| **Hepatopulmonary syndrome** | ICD-10 | K76.81 | Hepatopulmonary syndrome |
|  | ICD-9 | 573.5 | Hepatopulmonary syndrome |
| **HIV infection** | ICD-10 | B20 | HIV infection |
|  | ICD-9 | 042 | HIV infection |
| **Paget's disease** | ICD-10 | M88.0 | Osteitis deformans of skull |
|  | ICD-10 | M88.1 | Osteitis deformans of vertebrae |
|  | ICD-10 | M88.811 | Osteitis deformans of right shoulder |
|  | ICD-10 | M88.812 | Osteitis deformans of left shoulder |
|  | ICD-10 | M88.819 | Osteitis deformans of unspecified shoulder |
|  | ICD-10 | M88.821 | Osteitis deformans of right upper arm |
|  | ICD-10 | M88.822 | Osteitis deformans of left upper arm |
|  | ICD-10 | M88.829 | Osteitis deformans of unspecified upper arm |
|  | ICD-10 | M88.831 | Osteitis deformans of right forearm |
|  | ICD-10 | M88.832 | Osteitis deformans of left forearm |
|  | ICD-10 | M88.839 | Osteitis deformans of unspecified forearm |
|  | ICD-10 | M88.841 | Osteitis deformans of right hand |
|  | ICD-10 | M88.842 | Osteitis deformans of left hand |
|  | ICD-10 | M88.849 | Osteitis deformans of unspecified hand |
|  | ICD-10 | M88.851 | Osteitis deformans of right thigh |
|  | ICD-10 | M88.852 | Osteitis deformans of left thigh |
|  | ICD-10 | M88.859 | Osteitis deformans of unspecified thigh |
|  | ICD-10 | M88.861 | Osteitis deformans of right lower leg |
|  | ICD-10 | M88.862 | Osteitis deformans of left lower leg |
|  | ICD-10 | M88.869 | Osteitis deformans of unspecified lower leg |
|  | ICD-10 | M88.871 | Osteitis deformans of right ankle and foot |
|  | ICD-10 | M88.872 | Osteitis deformans of left ankle and foot |
|  | ICD-10 | M88.879 | Osteitis deformans of unspecified ankle and foot |
|  | ICD-10 | M88.88 | Osteitis deformans of other bones |
|  | ICD-10 | M88.89 | Osteitis deformans of multiple sites |
|  | ICD-10 | M88.9 | Osteitis deformans of unspecified bone |
|  | ICD-9 | 731.0 | Osteitis deformans without mention of bone tumor |
| **Fracture (within 3 months of cohort entry)** | ICD-10 | S02 | Fracture of skull and facial bones |
|  | ICD-10 | S12 | Fracture of cervical vertebra and other parts of neck |
|  | ICD-10 | S22 | Fracture of rib(s), sternum and thoracic spine |
|  | ICD-10 | S32 | Fracture of lumbar spine and pelvis |
|  | ICD-10 | S42 | Fracture of shoulder and upper arm |
|  | ICD-10 | S52 | Fracture of forearm |
|  | ICD-10 | S62 | Fracture at wrist and hand level |
|  | ICD-10 | S72 | Fracture of femur |
|  | ICD-10 | S82 | Fracture of lower leg, including ankle |
|  | ICD-10 | S92 | Fracture of foot and toe, except ankle |
|  | ICD-9 | 800 | Fracture of vault of skull |
|  | ICD-9 | 801 | Fracture of base of skull |
|  | ICD-9 | 802 | Fracture of face bones |
|  | ICD-9 | 803 | Other and unqualified skull fractures |
|  | ICD-9 | 804 | Multiple fractures involving skull or face with other bones |
|  | ICD-9 | 805 | Fracture of vertebral column without mention of spinal cord injury |
|  | ICD-9 | 806 | Fracture of vertebral column with spinal cord injury |
|  | ICD-9 | 807 | Fracture of rib(s) sternum larynx and trachea |
|  | ICD-9 | 808 | Fracture of pelvis |
|  | ICD-9 | 809 | Ill-defined fractures of bones of trunk |
|  | ICD-9 | 810 | Fracture of clavicle |
|  | ICD-9 | 811 | Fracture of scapula |
|  | ICD-9 | 812 | Fracture of humerus |
|  | ICD-9 | 813 | Fracture of radius and ulna |
|  | ICD-9 | 814 | Fracture of carpal bone(s) |
|  | ICD-9 | 815 | Fracture of metacarpal bone(s) |
|  | ICD-9 | 816 | Fracture of one or more phalanges of hand |
|  | ICD-9 | 817 | Multiple fractures of hand bones |
|  | ICD-9 | 818 | Ill-defined fractures of upper limb |
|  | ICD-9 | 819 | Multiple fractures involving both upper limbs and upper limb with rib(s) and sternum |
|  | ICD-9 | 820 | Fracture of neck of femur |
|  | ICD-9 | 821 | Fracture of other and unspecified parts of femur |
|  | ICD-9 | 822 | Fracture of patella |
|  | ICD-9 | 823 | Fracture of tibia and fibula |
|  | ICD-9 | 824 | Fracture of ankle |
|  | ICD-9 | 825 | Fracture of one or more tarsal and metatarsal bones |
|  | ICD-9 | 826 | Fracture of one or more phalanges of foot |
|  | ICD-9 | 827 | Other multiple and ill-defined fractures of lower limb |
|  | ICD-9 | 828 | Multiple fractures involving both lower limbs lower with upper limb and lower limb(s) with rib(s) and sternum |
|  | ICD-9 | 829 | Fracture of unspecified bones |
| **Liver transplant** | ICD-10 | Z48.23 | Encounter for aftercare following liver transplant |
|  | ICD-9 | V42.7 | Liver transplant status |
|  | ICD-10 | T86.40 | Unspecified complication of liver transplant |
|  | ICD-10 | T86.41 | Liver transplant rejection |
|  | ICD-10 | T86.42 | Liver transplant failure |
|  | ICD-10 | T86.43 | Liver transplant infection |
|  | ICD-10 | T86.49 | Other complications of liver transplant |
|  | ICD-9 | 996.82 | Complications of transplanted liver |
| **Obeticholic acid** | NDC | 69516001030 | OCA(gnrc_nm:Ocaliva) |
| **Obeticholic acid** | NDC | 69516000530 | OCA(Ocaliva) |
| **Fibrate** | NDC | 65862076990 | Fibrate |
|  | NDC | 62332053990 | Fibrate |
|  | NDC | 50268031211 | Fibrate |
|  | NDC | 62332055490 | Fibrate |
|  | NDC | 55700003130 | Fibrate |
|  | NDC | 00378608993 | Fibrate |
|  | NDC | 12280005190 | Fibrate |
|  | NDC | 27437010706 | Fibrate |
|  | NDC | 65726040110 | Fibrate |
|  | NDC | 00179010380 | Fibrate |
|  | NDC | 62332036190 | Fibrate |
|  | NDC | 13668043901 | Fibrate |
|  | NDC | 71205094972 | Fibrate |
|  | NDC | 54868557502 | Fibrate |
|  | NDC | 55111039590 | Fibrate |
|  | NDC | 27437010806 | Fibrate |
|  | NDC | 67707013099 | Fibrate |
|  | NDC | 27241011703 | Fibrate |
|  | NDC | 00378863077 | Fibrate |
|  | NDC | 50268031111 | Fibrate |
|  | NDC | 63187094090 | Fibrate |
|  | NDC | 31722059590 | Fibrate |
|  | NDC | 60760090130 | Fibrate |
|  | NDC | 68462058001 | Fibrate |
|  | NDC | 55700031990 | Fibrate |
|  | NDC | 51407009390 | Fibrate |
|  | NDC | 68382023016 | Fibrate |
|  | NDC | 00074317390 | Fibrate |
|  | NDC | 70756021590 | Fibrate |
|  | NDC | 51079060820 | Fibrate |
|  | NDC | 69097089607 | Fibrate |
|  | NDC | 50090289902 | Fibrate |
|  | NDC | 60760066030 | Fibrate |
|  | NDC | 68084032911 | Fibrate |
|  | NDC | 52725049590 | Fibrate |
|  | NDC | 00378306677 | Fibrate |
|  | NDC | 68084032811 | Fibrate |
|  | NDC | 00115152310 | Fibrate |
|  | NDC | 68180036009 | Fibrate |
|  | NDC | 63629109903 | Fibrate |
|  | NDC | 68382027016 | Fibrate |
|  | NDC | 43975030510 | Fibrate |
|  | NDC | 60760041130 | Fibrate |
|  | NDC | 51138051830 | Fibrate |
|  | NDC | 54868557501 | Fibrate |
|  | NDC | 50268031215 | Fibrate |
|  | NDC | 60505312003 | Fibrate |
|  | NDC | 51407009290 | Fibrate |
|  | NDC | 63629110101 | Fibrate |
|  | NDC | 60505312109 | Fibrate |
|  | NDC | 54569575000 | Fibrate |
|  | NDC | 35356090530 | Fibrate |
|  | NDC | 00093801101 | Fibrate |
|  | NDC | 68084063511 | Fibrate |
|  | NDC | 69367025709 | Fibrate |
|  | NDC | 00378608977 | Fibrate |
|  | NDC | 62332036071 | Fibrate |
|  | NDC | 16714074002 | Fibrate |
|  | NDC | 50268031012 | Fibrate |
|  | NDC | 68084082795 | Fibrate |
|  | NDC | 00378862977 | Fibrate |
|  | NDC | 54569575002 | Fibrate |
|  | NDC | 62559046190 | Fibrate |
|  | NDC | 27241012004 | Fibrate |
|  | NDC | 54868578400 | Fibrate |
|  | NDC | 54868557500 | Fibrate |
|  | NDC | 69367025409 | Fibrate |
|  | NDC | 63304090090 | Fibrate |
|  | NDC | 71205094978 | Fibrate |
|  | NDC | 00378710177 | Fibrate |
|  | NDC | 42385093605 | Fibrate |
|  | NDC | 51079059901 | Fibrate |
|  | NDC | 70756021451 | Fibrate |
|  | NDC | 35561034411 | Fibrate |
|  | NDC | 63304090105 | Fibrate |
|  | NDC | 16714074102 | Fibrate |
|  | NDC | 31722059690 | Fibrate |
|  | NDC | 50090325500 | Fibrate |
|  | NDC | 43598091090 | Fibrate |
|  | NDC | 54868551200 | Fibrate |
|  | NDC | 00378862877 | Fibrate |
|  | NDC | 68180036309 | Fibrate |
|  | NDC | 65862076890 | Fibrate |
|  | NDC | 52725049090 | Fibrate |
|  | NDC | 71205094955 | Fibrate |
|  | NDC | 71335074102 | Fibrate |
|  | NDC | 54868569700 | Fibrate |
|  | NDC | 72189017230 | Fibrate |
|  | NDC | 68682052501 | Fibrate |
|  | NDC | 62332036090 | Fibrate |
|  | NDC | 63304044430 | Fibrate |
|  | NDC | 00378439077 | Fibrate |
|  | NDC | 00904712804 | Fibrate |
|  | NDC | 71205094960 | Fibrate |
|  | NDC | 54868459500 | Fibrate |
|  | NDC | 00378306577 | Fibrate |
|  | NDC | 21695059330 | Fibrate |
|  | NDC | 71205094930 | Fibrate |
|  | NDC | 54569575001 | Fibrate |
|  | NDC | 42858020001 | Fibrate |
|  | NDC | 50090127500 | Fibrate |
|  | NDC | 50268031011 | Fibrate |
|  | NDC | 42858045445 | Fibrate |
|  | NDC | 68180036102 | Fibrate |
|  | NDC | 16714073902 | Fibrate |
|  | NDC | 43353024883 | Fibrate |
|  | NDC | 68084082711 | Fibrate |
|  | NDC | 27437011006 | Fibrate |
|  | NDC | 63187076030 | Fibrate |
|  | NDC | 60505312103 | Fibrate |
|  | NDC | 54868551201 | Fibrate |
|  | NDC | 71205019090 | Fibrate |
|  | NDC | 68462058101 | Fibrate |
|  | NDC | 68180036109 | Fibrate |
|  | NDC | 00074400990 | Fibrate |
|  | NDC | 42291029030 | Fibrate |
|  | NDC | 55700031930 | Fibrate |
|  | NDC | 27241011705 | Fibrate |
|  | NDC | 61269021090 | Fibrate |
|  | NDC | 13668044001 | Fibrate |
|  | NDC | 68084063625 | Fibrate |
|  | NDC | 67263004890 | Fibrate |
|  | NDC | 16714073801 | Fibrate |
|  | NDC | 60760090030 | Fibrate |
|  | NDC | 65726040125 | Fibrate |
|  | NDC | 35561034413 | Fibrate |
|  | NDC | 66869014720 | Fibrate |
|  | NDC | 58016004400 | Fibrate |
|  | NDC | 00378608893 | Fibrate |
|  | NDC | 55111034930 | Fibrate |
|  | NDC | 62332036171 | Fibrate |
|  | NDC | 63629109901 | Fibrate |
|  | NDC | 67707004330 | Fibrate |
|  | NDC | 43598090990 | Fibrate |
|  | NDC | 50268031112 | Fibrate |
|  | NDC | 68084063695 | Fibrate |
|  | NDC | 27437010906 | Fibrate |
|  | NDC | 63304044330 | Fibrate |
|  | NDC | 54868549800 | Fibrate |
|  | NDC | 66869014730 | Fibrate |
|  | NDC | 62332055591 | Fibrate |
|  | NDC | 13668043801 | Fibrate |
|  | NDC | 00093800901 | Fibrate |
|  | NDC | 71205036690 | Fibrate |
|  | NDC | 69367025405 | Fibrate |
|  | NDC | 54868566001 | Fibrate |
|  | NDC | 35356060330 | Fibrate |
|  | NDC | 00074612290 | Fibrate |
|  | NDC | 51138045830 | Fibrate |
|  | NDC | 21695059390 | Fibrate |
|  | NDC | 00378439177 | Fibrate |
|  | NDC | 00378863005 | Fibrate |
|  | NDC | 51138045730 | Fibrate |
|  | NDC | 49999088590 | Fibrate |
|  | NDC | 71205094990 | Fibrate |
|  | NDC | 54569470900 | Fibrate |
|  | NDC | 00179009180 | Fibrate |
|  | NDC | 68180013106 | Fibrate |
|  | NDC | 65726040325 | Fibrate |
|  | NDC | 58016004430 | Fibrate |
|  | NDC | 59630048590 | Fibrate |
|  | NDC | 68012049590 | Fibrate |
|  | NDC | 12280032815 | Fibrate |
|  | NDC | 43598091005 | Fibrate |
|  | NDC | 68180036209 | Fibrate |
|  | NDC | 50090304400 | Fibrate |
|  | NDC | 69097089507 | Fibrate |
|  | NDC | 69097045805 | Fibrate |
|  | NDC | 57844032401 | Fibrate |
|  | NDC | 51407009490 | Fibrate |
|  | NDC | 68382026916 | Fibrate |
|  | NDC | 51138045710 | Fibrate |
|  | NDC | 58016004460 | Fibrate |
|  | NDC | 43353003298 | Fibrate |
|  | NDC | 55289097330 | Fibrate |
|  | NDC | 63187076090 | Fibrate |
|  | NDC | 00378306605 | Fibrate |
|  | NDC | 50090337300 | Fibrate |
|  | NDC | 43353089819 | Fibrate |
|  | NDC | 51138045810 | Fibrate |
|  | NDC | 45802031575 | Fibrate |
|  | NDC | 54868522401 | Fibrate |
|  | NDC | 42291028990 | Fibrate |
|  | NDC | 51655001052 | Fibrate |
|  | NDC | 12280032830 | Fibrate |
|  | NDC | 50090269200 | Fibrate |
|  | NDC | 59630048030 | Fibrate |
|  | NDC | 54868520301 | Fibrate |
|  | NDC | 43547043109 | Fibrate |
|  | NDC | 68682052801 | Fibrate |
|  | NDC | 42291029290 | Fibrate |
|  | NDC | 00378862905 | Fibrate |
|  | NDC | 00115552210 | Fibrate |
|  | NDC | 63304044805 | Fibrate |
|  | NDC | 00093775598 | Fibrate |
|  | NDC | 68180038809 | Fibrate |
|  | NDC | 50090254600 | Fibrate |
|  | NDC | 45802013275 | Fibrate |
|  | NDC | 54868599401 | Fibrate |
|  | NDC | 42858006701 | Fibrate |
|  | NDC | 27241011904 | Fibrate |
|  | NDC | 35561034311 | Fibrate |
|  | NDC | 00115051101 | Fibrate |
|  | NDC | 63304044490 | Fibrate |
|  | NDC | 68180023109 | Fibrate |
|  | NDC | 69597034630 | Fibrate |
|  | NDC | 61269021290 | Fibrate |
|  | NDC | 70199002730 | Fibrate |
|  | NDC | 43353000119 | Fibrate |
|  | NDC | 63304044890 | Fibrate |
|  | NDC | 68180023209 | Fibrate |
|  | NDC | 42291029090 | Fibrate |
|  | NDC | 54868459700 | Fibrate |
|  | NDC | 59630049090 | Fibrate |
|  | NDC | 16714073701 | Fibrate |
|  | NDC | 70868001315 | Fibrate |
|  | NDC | 43975030610 | Fibrate |
|  | NDC | 43353027230 | Fibrate |
|  | NDC | 62332053991 | Fibrate |
|  | NDC | 68084032821 | Fibrate |
|  | NDC | 70199001330 | Fibrate |
|  | NDC | 42254034130 | Fibrate |
|  | NDC | 00093206098 | Fibrate |
|  | NDC | 00074644790 | Fibrate |
|  | NDC | 00093801201 | Fibrate |
|  | NDC | 43975044410 | Fibrate |
|  | NDC | 67707013030 | Fibrate |
|  | NDC | 00904681004 | Fibrate |
|  | NDC | 51407009190 | Fibrate |
|  | NDC | 57844032301 | Fibrate |
|  | NDC | 54868599400 | Fibrate |
|  | NDC | 00074641590 | Fibrate |
|  | NDC | 54569629900 | Fibrate |
|  | NDC | 00074434290 | Fibrate |
|  | NDC | 00074612390 | Fibrate |
|  | NDC | 50090285700 | Fibrate |
|  | NDC | 67544103398 | Fibrate |
|  | NDC | 00074318990 | Fibrate |
|  | NDC | 00378306505 | Fibrate |
|  | NDC | 57844032201 | Fibrate |
|  | NDC | 54868566000 | Fibrate |
|  | NDC | 43353003283 | Fibrate |
|  | NDC | 66869013720 | Fibrate |
|  | NDC | 51079059920 | Fibrate |
|  | NDC | 51407009090 | Fibrate |
|  | NDC | 60760031830 | Fibrate |
|  | NDC | 69097045905 | Fibrate |
|  | NDC | 68084082721 | Fibrate |
|  | NDC | 71610002783 | Fibrate |
|  | NDC | 00115052202 | Fibrate |
|  | NDC | 62332053890 | Fibrate |
|  | NDC | 43353027283 | Fibrate |
|  | NDC | 50268030911 | Fibrate |
|  | NDC | 50268031311 | Fibrate |
|  | NDC | 62332055590 | Fibrate |
|  | NDC | 27241011804 | Fibrate |
|  | NDC | 00074401390 | Fibrate |
|  | NDC | 68084063521 | Fibrate |
|  | NDC | 68012049090 | Fibrate |
|  | NDC | 70756021490 | Fibrate |
|  | NDC | 68180038902 | Fibrate |
|  | NDC | 42858013401 | Fibrate |
|  | NDC | 50090305600 | Fibrate |
|  | NDC | 00115053302 | Fibrate |
|  | NDC | 27241011603 | Fibrate |
|  | NDC | 66869013730 | Fibrate |
|  | NDC | 43547043009 | Fibrate |
|  | NDC | 00115551110 | Fibrate |
|  | NDC | 68382022816 | Fibrate |
|  | NDC | 00115052201 | Fibrate |
|  | NDC | 55111039530 | Fibrate |
|  | NDC | 63629109904 | Fibrate |
|  | NDC | 63304044905 | Fibrate |
|  | NDC | 68180038909 | Fibrate |
|  | NDC | 71205005090 | Fibrate |
|  | NDC | 67263026790 | Fibrate |
|  | NDC | 42858066045 | Fibrate |
|  | NDC | 63629110001 | Fibrate |
|  | NDC | 54868522400 | Fibrate |
|  | NDC | 50090127501 | Fibrate |
|  | NDC | 00115552202 | Fibrate |
|  | NDC | 54868520300 | Fibrate |
|  | NDC | 68084083532 | Fibrate |
|  | NDC | 51138045610 | Fibrate |
|  | NDC | 27437011001 | Fibrate |
|  | NDC | 65726040310 | Fibrate |
|  | NDC | 50268031315 | Fibrate |
|  | NDC | 54868454100 | Fibrate |
|  | NDC | 63304044990 | Fibrate |
|  | NDC | 68084082725 | Fibrate |
|  | NDC | 51138051930 | Fibrate |
|  | NDC | 12280032890 | Fibrate |
|  | NDC | 59630048090 | Fibrate |
|  | NDC | 51079060801 | Fibrate |
|  | NDC | 00115152210 | Fibrate |
|  | NDC | 00378710077 | Fibrate |
|  | NDC | 00115053301 | Fibrate |
|  | NDC | 70756021551 | Fibrate |
|  | NDC | 63304090190 | Fibrate |
|  | NDC | 51138045630 | Fibrate |
|  | NDC | 00093775698 | Fibrate |
|  | NDC | 42385093690 | Fibrate |
|  | NDC | 68462058201 | Fibrate |
|  | NDC | 57844069298 | Fibrate |
|  | NDC | 68084032921 | Fibrate |
|  | NDC | 49999088530 | Fibrate |
|  | NDC | 54868611800 | Fibrate |
|  | NDC | 59630049590 | Fibrate |
|  | NDC | 57844069198 | Fibrate |
|  | NDC | 72189002630 | Fibrate |
|  | NDC | 62559046090 | Fibrate |
|  | NDC | 59630048530 | Fibrate |
|  | NDC | 58016004490 | Fibrate |
|  | NDC | 00093206198 | Fibrate |
|  | NDC | 69097089407 | Fibrate |
|  | NDC | 68084083533 | Fibrate |
|  | NDC | 68180013006 | Fibrate |
|  | NDC | 54868569701 | Fibrate |
|  | NDC | 42385093590 | Fibrate |
|  | NDC | 50268030913 | Fibrate |
| **Spironolactone** | NDC | 00025100151 | Spironolactone |
|  | NDC | 00677170807 | Spironolactone |
|  | NDC | 16729022616 | Spironolactone |
|  | NDC | 53489032805 | Spironolactone |
|  | NDC | 16714008602 | Spironolactone |
|  | NDC | 53489032901 | Spironolactone |
|  | NDC | 00228267350 | Spironolactone |
|  | NDC | 53746051110 | Spironolactone |
|  | NDC | 51079097901 | Spironolactone |
|  | NDC | 59762501201 | Spironolactone |
|  | NDC | 00440838592 | Spironolactone |
|  | NDC | 54868501501 | Spironolactone |
|  | NDC | 62991121103 | Spironolactone, micronized |
|  | NDC | 65162051510 | Spironolactone |
|  | NDC | 51079010319 | Spironolactone |
|  | NDC | 60687046511 | Spironolactone |
|  | NDC | 63739054510 | Spironolactone |
|  | NDC | 67544040545 | Spironolactone |
|  | NDC | 00904692761 | Spironolactone |
|  | NDC | 59746021601 | Spironolactone |
|  | NDC | 63370022425 | Spironolactone, micronized |
|  | NDC | 53746051101 | Spironolactone |
|  | NDC | 00603576328 | Spironolactone |
|  | NDC | 51552027605 | Spironolactone |
|  | NDC | 00781159901 | Spironolactone |
|  | NDC | 00677170705 | Spironolactone |
|  | NDC | 68084020611 | Spironolactone |
|  | NDC | 68084020701 | Spironolactone |
|  | NDC | 38779009605 | Spironolactone |
|  | NDC | 53746051401 | Spironolactone |
|  | NDC | 63739041610 | Spironolactone |
|  | NDC | 00677062507 | Spironolactone |
|  | NDC | 49452722002 | Spironolactone |
|  | NDC | 68382066201 | Spironolactone |
|  | NDC | 53746051505 | Spironolactone |
|  | NDC | 67544040560 | Spironolactone |
|  | NDC | 52372064102 | Spironolactone |
|  | NDC | 16714008501 | Spironolactone |
|  | NDC | 53489014305 | Spironolactone |
|  | NDC | 65162051111 | Spironolactone |
|  | NDC | 59762501102 | Spironolactone |
|  | NDC | 00228280311 | Spironolactone |
|  | NDC | 16729022501 | Spironolactone |
|  | NDC | 00781159910 | Spironolactone |
|  | NDC | 00603576521 | Spironolactone |
|  | NDC | 67544031080 | Spironolactone |
|  | NDC | 63629106501 | Spironolactone |
|  | NDC | 54868070000 | Spironolactone |
|  | NDC | 16714008402 | Spironolactone |
|  | NDC | 00677062505 | Spironolactone |
|  | NDC | 54868070005 | Spironolactone |
|  | NDC | 51927137700 | Spironolactone |
|  | NDC | 16729022517 | Spironolactone |
|  | NDC | 55289050730 | Spironolactone |
|  | NDC | 00904595206 | Spironolactone |
|  | NDC | 68382066010 | Spironolactone |
|  | NDC | 63629106701 | Spironolactone |
|  | NDC | 00025104131 | Spironolactone |
|  | NDC | 00677170805 | Spironolactone |
|  | NDC | 62991121104 | Spironolactone, micronized |
|  | NDC | 67544031015 | Spironolactone |
|  | NDC | 53489032807 | Spironolactone |
|  | NDC | 68382066105 | Spironolactone |
|  | NDC | 00378214605 | Spironolactone |
|  | NDC | 54868447702 | Spironolactone |
|  | NDC | 00025104134 | Spironolactone |
|  | NDC | 63629106402 | Spironolactone |
|  | NDC | 00025100131 | Spironolactone |
|  | NDC | 00677170707 | Spironolactone |
|  | NDC | 00378024305 | Spironolactone |
|  | NDC | 67544031030 | Spironolactone |
|  | NDC | 53746051105 | Spironolactone |
|  | NDC | 58864067328 | Spironolactone |
|  | NDC | 60687046501 | Spironolactone |
|  | NDC | 46287002004 | Spironolactone |
|  | NDC | 63739054410 | Spironolactone |
|  | NDC | 55289050701 | Spironolactone |
|  | NDC | 68115042330 | Spironolactone |
|  | NDC | 51079010320 | Spironolactone |
|  | NDC | 53489032906 | Spironolactone |
|  | NDC | 54738013101 | Spironolactone |
|  | NDC | 16729022701 | Spironolactone |
|  | NDC | 50090272800 | Spironolactone |
|  | NDC | 16729022716 | Spironolactone |
|  | NDC | 53489032801 | Spironolactone |
|  | NDC | 00378014101 | Spironolactone/HCTZ |
|  | NDC | 59762501301 | Spironolactone |
|  | NDC | 54868501500 | Spironolactone |
|  | NDC | 46287002001 | Spironolactone |
|  | NDC | 00904595161 | Spironolactone |
|  | NDC | 68382066001 | Spironolactone |
|  | NDC | 68084020801 | Spironolactone |
|  | NDC | 63629109401 | Spironolactone |
|  | NDC | 71610028745 | Spironolactone |
|  | NDC | 51079098001 | Spironolactone |
|  | NDC | 00440838560 | Spironolactone |
|  | NDC | 43353023960 | Spironolactone |
|  | NDC | 51552027602 | Spironolactone |
|  | NDC | 00228267211 | Spironolactone |
|  | NDC | 65162051550 | Spironolactone |
|  | NDC | 00603576428 | Spironolactone |
|  | NDC | 51079010317 | Spironolactone |
|  | NDC | 49452722003 | Spironolactone |
|  | NDC | 51079098008 | Spironolactone |
|  | NDC | 67544031060 | Spironolactone |
|  | NDC | 60687048701 | Spironolactone |
|  | NDC | 00603576332 | Spironolactone |
|  | NDC | 68382066205 | Spironolactone |
|  | NDC | 52372064103 | Spironolactone |
|  | NDC | 00677170701 | Spironolactone |
|  | NDC | 00378043701 | Spironolactone |
|  | NDC | 10544049990 | Spironolactone |
|  | NDC | 65162051110 | Spironolactone |
|  | NDC | 54868070006 | Spironolactone |
|  | NDC | 65162051410 | Spironolactone |
|  | NDC | 51552027601 | Spironolactone |
|  | NDC | 54868447701 | Spironolactone |
|  | NDC | 59746021605 | Spironolactone |
|  | NDC | 67544031045 | Spironolactone |
|  | NDC | 00440838530 | Spironolactone |
|  | NDC | 16714008603 | Spironolactone |
|  | NDC | 59746021701 | Spironolactone |
|  | NDC | 63739054404 | Spironolactone |
|  | NDC | 00677170806 | Spironolactone |
|  | NDC | 54738013003 | Spironolactone |
|  | NDC | 51079098020 | Spironolactone |
|  | NDC | 50268072611 | Spironolactone |
|  | NDC | 38779009603 | Spironolactone |
|  | NDC | 52372064101 | Spironolactone |
|  | NDC | 63629106101 | Spironolactone |
|  | NDC | 54738013102 | Spironolactone |
|  | NDC | 51552027604 | Spironolactone |
|  | NDC | 53489032905 | Spironolactone |
|  | NDC | 62991121102 | Spironolactone, micronized |
|  | NDC | 54868308700 | Spironolactone |
|  | NDC | 00228267250 | Spironolactone |
|  | NDC | 49452722001 | Spironolactone |
|  | NDC | 65162051150 | Spironolactone |
|  | NDC | 16714008403 | Spironolactone |
|  | NDC | 60429022960 | Spironolactone |
|  | NDC | 00228280350 | Spironolactone |
|  | NDC | 00603576330 | Spironolactone |
|  | NDC | 63629106403 | Spironolactone |
|  | NDC | 54868070001 | Spironolactone |
|  | NDC | 00677170706 | Spironolactone |
|  | NDC | 16714008401 | Spironolactone |
|  | NDC | 00677062506 | Spironolactone |
|  | NDC | 38779009604 | Spironolactone |
|  | NDC | 53489014301 | Spironolactone |
|  | NDC | 71610028730 | Spironolactone |
|  | NDC | 00603576421 | Spironolactone |
|  | NDC | 63629106401 | Spironolactone |
|  | NDC | 68382066101 | Spironolactone |
|  | NDC | 65162051450 | Spironolactone |
|  | NDC | 16714008604 | Spironolactone |
|  | NDC | 00603576528 | Spironolactone |
|  | NDC | 51927137700 | Spironolactone, micronized |
|  | NDC | 00228267311 | Spironolactone |
|  | NDC | 53489032907 | Spironolactone |
|  | NDC | 51079010363 | Spironolactone |
|  | NDC | 60760077190 | Spironolactone |
|  | NDC | 16729022601 | Spironolactone |
|  | NDC | 53746051501 | Spironolactone |
|  | NDC | 68084020601 | Spironolactone |
|  | NDC | 51079097920 | Spironolactone |
|  | NDC | 53746051405 | Spironolactone |
|  | NDC | 53489014310 | Spironolactone |
|  | NDC | 68084020711 | Spironolactone |
|  | NDC | 38779009601 | Spironolactone |
|  | NDC | 00781159905 | Spironolactone |
|  | NDC | 00677062501 | Spironolactone |
|  | NDC | 63739022610 | Spironolactone |
|  | NDC | 00378024301 | Spironolactone |
|  | NDC | 67544040530 | Spironolactone |
|  | NDC | 59762501101 | Spironolactone |
|  | NDC | 16714008502 | Spironolactone |
|  | NDC | 00025103134 | Spironolactone |
|  | NDC | 16729022516 | Spironolactone |
|  | NDC | 00603576321 | Spironolactone |
|  | NDC | 51079010301 | Spironolactone |
|  | NDC | 00677062510 | Spironolactone |
|  | NDC | 59746021801 | Spironolactone |
|  | NDC | 00025103131 | Spironolactone |
|  | NDC | 00378214601 | Spironolactone |
|  | NDC | 60687047601 | Spironolactone |
|  | NDC | 00378024393 | Spironolactone |
|  | NDC | 00677170801 | Spironolactone |
|  | NDC | 68382066005 | Spironolactone |
|  | NDC | 68084020811 | Spironolactone |
|  | NDC | 53489032806 | Spironolactone |
|  | NDC | 00440838591 | Spironolactone |
|  | NDC | 67544031053 | Spironolactone |
|  | NDC | 00904595306 | Spironolactone |
|  | NDC | 54569050501 | Spironolactone |
|  | NDC | 00603576516 | Spironolactone |
|  | NDC | 43063027730 | Spironolactone |
|  | NDC | 54738013001 | Spironolactone |
|  | NDC | 16714008601 | Spironolactone |
| **Furosemide** | NDC | 71610030980 | Furosemide |
|  | NDC | 63323028003 | Furosemide |
|  | NDC | 69315011710 | Furosemide |
|  | NDC | 00378020801 | Furosemide |
|  | NDC | 00781196610 | Furosemide |
|  | NDC | 23490562403 | Furosemide |
|  | NDC | 00409610225 | Furosemide |
|  | NDC | 43547040210 | Furosemide |
|  | NDC | 23155047344 | Furosemide |
|  | NDC | 51407011390 | Furosemide |
|  | NDC | 51079007301 | Furosemide |
|  | NDC | 51079007256 | Furosemide |
|  | NDC | 50742010401 | Furosemide |
|  | NDC | 00054429925 | Furosemide |
|  | NDC | 00054429931 | Furosemide |
|  | NDC | 55289059301 | Furosemide |
|  | NDC | 00603374104 | Furosemide |
|  | NDC | 68084001411 | Furosemide |
|  | NDC | 67253054110 | Furosemide |
|  | NDC | 54868005803 | Furosemide |
|  | NDC | 55150032325 | Furosemide |
|  | NDC | 00591343805 | Furosemide |
|  | NDC | 71610030860 | Furosemide |
|  | NDC | 54868078800 | Furosemide |
|  | NDC | 67544024430 | Furosemide |
|  | NDC | 00440755604 | Furosemide |
|  | NDC | 00440755560 | Furosemide |
|  | NDC | 00781181801 | Furosemide |
|  | NDC | 00904579861 | Furosemide |
|  | NDC | 58016053930 | Furosemide |
|  | NDC | 00039006070 | Furosemide |
|  | NDC | 54868005806 | Furosemide |
|  | NDC | 69315011701 | Furosemide |
|  | NDC | 00378020810 | Furosemide |
|  | NDC | 62991280601 | Furosemide |
|  | NDC | 71610004960 | Furosemide |
|  | NDC | 00409963104 | Furosemide |
|  | NDC | 21695049100 | Furosemide |
|  | NDC | 50742010410 | Furosemide |
|  | NDC | 67253054011 | Furosemide |
|  | NDC | 43353022130 | Furosemide |
|  | NDC | 23155047341 | Furosemide |
|  | NDC | 67544022053 | Furosemide |
|  | NDC | 00409610220 | Furosemide |
|  | NDC | 00781181810 | Furosemide |
|  | NDC | 67544007545 | Furosemide |
|  | NDC | 55045121708 | Furosemide |
|  | NDC | 23490562301 | Furosemide |
|  | NDC | 00781196601 | Furosemide |
|  | NDC | 51079007357 | Furosemide |
|  | NDC | 51079007220 | Furosemide |
|  | NDC | 49999022560 | Furosemide |
|  | NDC | 00172290710 | Furosemide |
|  | NDC | 00603373934 | Furosemide |
|  | NDC | 00039006710 | Furosemide |
|  | NDC | 21695049030 | Furosemide |
|  | NDC | 51079052719 | Furosemide |
|  | NDC | 64125011610 | Furosemide |
|  | NDC | 00054829925 | Furosemide |
|  | NDC | 55289059330 | Furosemide |
|  | NDC | 63323028026 | Furosemide |
|  | NDC | 51079007330 | Furosemide |
|  | NDC | 68094074262 | Furosemide |
|  | NDC | 00172290880 | Furosemide |
|  | NDC | 00039006050 | Furosemide |
|  | NDC | 71610030845 | Furosemide |
|  | NDC | 00378020893 | Furosemide |
|  | NDC | 68094074259 | Furosemide |
|  | NDC | 00440755792 | Furosemide |
|  | NDC | 00247075730 | Furosemide |
|  | NDC | 00591030205 | Furosemide |
|  | NDC | 68094075662 | Furosemide |
|  | NDC | 00054329450 | Furosemide |
|  | NDC | 23155047332 | Furosemide |
|  | NDC | 23629012810 | Furosemide |
|  | NDC | 43353074730 | Furosemide |
|  | NDC | 00603374121 | Furosemide |
|  | NDC | 00409163910 | Furosemide |
|  | NDC | 67544007560 | Furosemide |
|  | NDC | 64125011601 | Furosemide |
|  | NDC | 00603374034 | Furosemide |
|  | NDC | 63739011203 | Furosemide |
|  | NDC | 36000006505 | Furosemide |
|  | NDC | 36000028425 | Furosemide |
|  | NDC | 63304062601 | Furosemide |
|  | NDC | 49999003000 | Furosemide |
|  | NDC | 58016057603 | Furosemide |
|  | NDC | 43353006960 | Furosemide |
|  | NDC | 55150032201 | Furosemide |
|  | NDC | 43353073753 | Furosemide |
|  | NDC | 54868005704 | Furosemide |
|  | NDC | 55289011804 | Furosemide |
|  | NDC | 00039006605 | Furosemide |
|  | NDC | 64679075901 | Furosemide |
|  | NDC | 67544022092 | Furosemide |
|  | NDC | 63739011201 | Furosemide |
|  | NDC | 67544064960 | Furosemide |
|  | NDC | 43353073745 | Furosemide |
|  | NDC | 69315011801 | Furosemide |
|  | NDC | 43353074780 | Furosemide |
|  | NDC | 00591343610 | Furosemide |
|  | NDC | 67544056860 | Furosemide |
|  | NDC | 10019001076 | Furosemide |
|  | NDC | 43547040111 | Furosemide |
|  | NDC | 71610004953 | Furosemide |
|  | NDC | 63739011215 | Furosemide |
|  | NDC | 67544022060 | Furosemide |
|  | NDC | 71610009630 | Furosemide |
|  | NDC | 67544007592 | Furosemide |
|  | NDC | 21695049190 | Furosemide |
|  | NDC | 00603374132 | Furosemide |
|  | NDC | 00024060925 | Furosemide |
|  | NDC | 00378021693 | Furosemide |
|  | NDC | 00039006750 | Furosemide |
|  | NDC | 51079007219 | Furosemide |
|  | NDC | 54738093401 | Furosemide |
|  | NDC | 68084001701 | Furosemide |
|  | NDC | 54569057400 | Furosemide |
|  | NDC | 36000028325 | Furosemide |
|  | NDC | 68387036530 | Furosemide |
|  | NDC | 63739011210 | Furosemide |
|  | NDC | 51079052720 | Furosemide |
|  | NDC | 71610030853 | Furosemide |
|  | NDC | 00054430129 | Furosemide |
|  | NDC | 00054329446 | Furosemide |
|  | NDC | 63323028004 | Furosemide |
|  | NDC | 63323028010 | Furosemide |
|  | NDC | 00378021601 | Furosemide |
|  | NDC | 00440755520 | Furosemide |
|  | NDC | 49999017930 | Furosemide |
|  | NDC | 49999003090 | Furosemide |
|  | NDC | 00517570225 | Furosemide |
|  | NDC | 67544022045 | Furosemide |
|  | NDC | 43353073760 | Furosemide |
|  | NDC | 00409610202 | Furosemide |
|  | NDC | 54868005703 | Furosemide |
|  | NDC | 68084001611 | Furosemide |
|  | NDC | 00440755790 | Furosemide |
|  | NDC | 00904579761 | Furosemide |
|  | NDC | 00591343601 | Furosemide |
|  | NDC | 50742010601 | Furosemide |
|  | NDC | 00172290810 | Furosemide |
|  | NDC | 00247019900 | Furosemide |
|  | NDC | 52959075130 | Furosemide |
|  | NDC | 51079007317 | Furosemide |
|  | NDC | 63323028001 | Furosemide |
|  | NDC | 58517040030 | Furosemide |
|  | NDC | 43353022060 | Furosemide |
|  | NDC | 00378023205 | Furosemide |
|  | NDC | 00781144605 | Furosemide |
|  | NDC | 00039006340 | Furosemide |
|  | NDC | 43353022180 | Furosemide |
|  | NDC | 00591030110 | Furosemide |
|  | NDC | 55150032425 | Furosemide |
|  | NDC | 71610030930 | Furosemide |
|  | NDC | 63304062401 | Furosemide |
|  | NDC | 64125011805 | Furosemide |
|  | NDC | 63739054201 | Furosemide |
|  | NDC | 00409610219 | Furosemide |
|  | NDC | 51407011490 | Furosemide |
|  | NDC | 00409610236 | Furosemide |
|  | NDC | 43353006953 | Furosemide |
|  | NDC | 00409127532 | Furosemide |
|  | NDC | 67544007553 | Furosemide |
|  | NDC | 50742010510 | Furosemide |
|  | NDC | 00247075700 | Furosemide |
|  | NDC | 67544024492 | Furosemide |
|  | NDC | 63739011101 | Furosemide |
|  | NDC | 00182116189 | Furosemide |
|  | NDC | 54868005802 | Furosemide |
|  | NDC | 63304062410 | Furosemide |
|  | NDC | 51655008124 | Furosemide |
|  | NDC | 00039006770 | Furosemide |
|  | NDC | 23490562202 | Furosemide |
|  | NDC | 60432061360 | Furosemide |
|  | NDC | 00378021610 | Furosemide |
|  | NDC | 00591030101 | Furosemide |
|  | NDC | 00409610227 | Furosemide |
|  | NDC | 00172290780 | Furosemide |
|  | NDC | 54868078802 | Furosemide |
|  | NDC | 63739054210 | Furosemide |
|  | NDC | 55887099730 | Furosemide |
|  | NDC | 49999017960 | Furosemide |
|  | NDC | 00409610235 | Furosemide |
|  | NDC | 55150032225 | Furosemide |
|  | NDC | 30698006605 | Furosemide |
|  | NDC | 50742010501 | Furosemide |
|  | NDC | 00591343801 | Furosemide |
|  | NDC | 68084001401 | Furosemide |
|  | NDC | 71610030580 | Furosemide |
|  | NDC | 43353073730 | Furosemide |
|  | NDC | 43547040310 | Furosemide |
|  | NDC | 49999017990 | Furosemide |
|  | NDC | 00054430125 | Furosemide |
|  | NDC | 30698006701 | Furosemide |
|  | NDC | 38779022604 | Furosemide |
|  | NDC | 69315011601 | Furosemide |
|  | NDC | 43353074753 | Furosemide |
|  | NDC | 43547040211 | Furosemide |
|  | NDC | 63739011115 | Furosemide |
|  | NDC | 63323028016 | Furosemide |
|  | NDC | 63323028002 | Furosemide |
|  | NDC | 51079007257 | Furosemide |
|  | NDC | 67253054111 | Furosemide |
|  | NDC | 43353022030 | Furosemide |
|  | NDC | 71610004980 | Furosemide |
|  | NDC | 63739054101 | Furosemide |
|  | NDC | 54569057200 | Furosemide |
|  | NDC | 43353006980 | Furosemide |
|  | NDC | 23155047333 | Furosemide |
|  | NDC | 64125011701 | Furosemide |
|  | NDC | 55289011814 | Furosemide |
|  | NDC | 00182117000 | Furosemide |
|  | NDC | 58016053900 | Furosemide |
|  | NDC | 00039006711 | Furosemide |
|  | NDC | 55887097890 | Furosemide |
|  | NDC | 21695049130 | Furosemide |
|  | NDC | 49452322201 | Furosemide |
|  | NDC | 63739054104 | Furosemide |
|  | NDC | 71610030945 | Furosemide |
|  | NDC | 00409610204 | Furosemide |
|  | NDC | 00440755692 | Furosemide |
|  | NDC | 00039006013 | Furosemide |
|  | NDC | 71610009660 | Furosemide |
|  | NDC | 00172290700 | Furosemide |
|  | NDC | 51407011401 | Furosemide |
|  | NDC | 51079007230 | Furosemide |
|  | NDC | 00247075720 | Furosemide |
|  | NDC | 67253054010 | Furosemide |
|  | NDC | 71610030960 | Furosemide |
|  | NDC | 00517570425 | Furosemide |
|  | NDC | 71610030880 | Furosemide |
|  | NDC | 51079007201 | Furosemide |
|  | NDC | 69315011610 | Furosemide |
|  | NDC | 51079052717 | Furosemide |
|  | NDC | 51079007356 | Furosemide |
|  | NDC | 58016053830 | Furosemide |
|  | NDC | 00182116100 | Furosemide |
|  | NDC | 00517571025 | Furosemide |
|  | NDC | 63739011110 | Furosemide |
|  | NDC | 54569057207 | Furosemide |
|  | NDC | 36000028225 | Furosemide |
|  | NDC | 43353006930 | Furosemide |
|  | NDC | 51079007319 | Furosemide |
|  | NDC | 00054429725 | Furosemide |
|  | NDC | 54738093501 | Furosemide |
|  | NDC | 00603373932 | Furosemide |
|  | NDC | 58864022190 | Furosemide |
|  | NDC | 51407011310 | Furosemide |
|  | NDC | 68084001711 | Furosemide |
|  | NDC | 63739011303 | Furosemide |
|  | NDC | 36000006405 | Furosemide |
|  | NDC | 43063041679 | Furosemide |
|  | NDC | 67544007580 | Furosemide |
|  | NDC | 67544024453 | Furosemide |
|  | NDC | 67544007594 | Furosemide |
|  | NDC | 63304062605 | Furosemide |
|  | NDC | 55150032301 | Furosemide |
|  | NDC | 58864022030 | Furosemide |
|  | NDC | 67253054250 | Furosemide |
|  | NDC | 51407011410 | Furosemide |
|  | NDC | 00603373921 | Furosemide |
|  | NDC | 67544064980 | Furosemide |
|  | NDC | 67544022094 | Furosemide |
|  | NDC | 49999022530 | Furosemide |
|  | NDC | 00039006650 | Furosemide |
|  | NDC | 51407011301 | Furosemide |
|  | NDC | 00591343710 | Furosemide |
|  | NDC | 67544022030 | Furosemide |
|  | NDC | 64125011710 | Furosemide |
|  | NDC | 51079007320 | Furosemide |
|  | NDC | 51407011505 | Furosemide |
|  | NDC | 00054830125 | Furosemide |
|  | NDC | 43353006909 | Furosemide |
|  | NDC | 72789003330 | Furosemide |
|  | NDC | 00440755506 | Furosemide |
|  | NDC | 63739054110 | Furosemide |
|  | NDC | 63323028036 | Furosemide |
|  | NDC | 00172290860 | Furosemide |
|  | NDC | 00409610210 | Furosemide |
|  | NDC | 00182117089 | Furosemide |
|  | NDC | 00603374021 | Furosemide |
|  | NDC | 60432061304 | Furosemide |
|  | NDC | 00591030201 | Furosemide |
|  | NDC | 23490562302 | Furosemide |
|  | NDC | 51079007217 | Furosemide |
|  | NDC | 00054829816 | Furosemide |
|  | NDC | 51927178400 | Furosemide |
|  | NDC | 00781144601 | Furosemide |
|  | NDC | 00378023201 | Furosemide |
|  | NDC | 63739054310 | Furosemide |
|  | NDC | 43353022160 | Furosemide |
|  | NDC | 00054329863 | Furosemide |
|  | NDC | 43547040350 | Furosemide |
|  | NDC | 66336076130 | Furosemide |
|  | NDC | 71610030830 | Furosemide |
|  | NDC | 64125011801 | Furosemide |
|  | NDC | 54868218000 | Furosemide |
|  | NDC | 00039006306 | Furosemide |
|  | NDC | 30698006001 | Furosemide |
|  | NDC | 23490562203 | Furosemide |
|  | NDC | 64679075902 | Furosemide |
|  | NDC | 68084001485 | Furosemide |
|  | NDC | 50742010605 | Furosemide |
|  | NDC | 36000006305 | Furosemide |
|  | NDC | 63739011310 | Furosemide |
|  | NDC | 67544007530 | Furosemide |
|  | NDC | 00054429731 | Furosemide |
|  | NDC | 43547040110 | Furosemide |
|  | NDC | 54868218800 | Furosemide |
|  | NDC | 68084001601 | Furosemide |
|  | NDC | 00904579661 | Furosemide |
|  | NDC | 00378023293 | Furosemide |
|  | NDC | 00591343701 | Furosemide |
|  | NDC | 71610030953 | Furosemide |
|  | NDC | 00074963104 | Furosemide |
|  | NDC | 67253054210 | Furosemide |
|  | NDC | 00039006011 | Furosemide |
|  | NDC | 23155047331 | Furosemide |
|  | NDC | 00247019930 | Furosemide |
|  | NDC | 63739011315 | Furosemide |
|  | NDC | 67544022080 | Furosemide |
|  | NDC | 63739011301 | Furosemide |
|  | NDC | 67544024445 | Furosemide |
|  | NDC | 69315011805 | Furosemide |
|  | NDC | 00603374032 | Furosemide |
|  | NDC | 43353074760 | Furosemide |
|  | NDC | 00591030001 | Furosemide |
|  | NDC | 00409610218 | Furosemide |
|  | NDC | 71610009653 | Furosemide |
|  | NDC | 51079052701 | Furosemide |
|  | NDC | 23155047342 | Furosemide |
|  | NDC | 63304062510 | Furosemide |
|  | NDC | 00172290760 | Furosemide |
|  | NDC | 71610004930 | Furosemide |
|  | NDC | 00054829725 | Furosemide |
|  | NDC | 00603374102 | Furosemide |
|  | NDC | 00172290800 | Furosemide |
|  | NDC | 00409610226 | Furosemide |
|  | NDC | 43353074745 | Furosemide |
|  | NDC | 63304062501 | Furosemide |
|  | NDC | 00591030010 | Furosemide |
|  | NDC | 66116023630 | Furosemide |
|  | NDC | 00440755607 | Furosemide |
|  | NDC | 43353073780 | Furosemide |
|  | NDC | 00603374128 | Furosemide |
|  | NDC | 67544024460 | Furosemide |
|  | NDC | 63739011103 | Furosemide |
| **Lactulose** | NDC | 62559550108 | Lactulose |
|  | NDC | 00121057708 | Lactulose |
|  | NDC | 39328054132 | Lactulose |
|  | NDC | 00121115440 | Lactulose |
|  | NDC | 66689003950 | Lactulose |
|  | NDC | 63739053855 | Lactulose |
|  | NDC | 66220072930 | Lactulose |
|  | NDC | 50383077917 | Lactulose |
|  | NDC | 00121457740 | Lactulose |
|  | NDC | 42769134008 | Lactulose |
|  | NDC | 00603137865 | Lactulose |
|  | NDC | 00121057716 | Lactulose |
|  | NDC | 00591251965 | Lactulose |
|  | NDC | 60432003816 | Lactulose |
|  | NDC | 54868310101 | Lactulose |
|  | NDC | 00591234716 | Lactulose |
|  | NDC | 60505036001 | Lactulose |
|  | NDC | 00054348663 | Lactulose |
|  | NDC | 00472500159 | Lactulose |
|  | NDC | 62794050293 | Lactulose |
|  | NDC | 00054848616 | Lactulose |
|  | NDC | 50383077932 | Lactulose |
|  | NDC | 00121087316 | Lactulose |
|  | NDC | 50383077930 | Lactulose |
|  | NDC | 60432003708 | Lactulose |
|  | NDC | 13668057408 | Lactulose |
|  | NDC | 00472500160 | Lactulose |
|  | NDC | 45963043963 | Lactulose |
|  | NDC | 50383077915 | Lactulose |
|  | NDC | 00603137856 | Lactulose |
|  | NDC | 54868530000 | Lactulose |
|  | NDC | 00121087308 | Lactulose |
|  | NDC | 00068041316 | Lactulose |
|  | NDC | 00603137859 | Lactulose |
|  | NDC | 66689003901 | Lactulose |
|  | NDC | 68115062616 | Lactulose |
|  | NDC | 42769134003 | Lactulose |
|  | NDC | 13668057410 | Lactulose |
|  | NDC | 45963043965 | Lactulose |
|  | NDC | 00591234764 | Lactulose |
|  | NDC | 00182607297 | Lactulose |
|  | NDC | 00472136016 | Lactulose |
|  | NDC | 62794050217 | Lactulose |
|  | NDC | 00839719969 | Lactulose |
|  | NDC | 66689003850 | Lactulose |
|  | NDC | 58165000316 | Lactulose |
|  | NDC | 00121057732 | Lactulose |
|  | NDC | 66220071930 | Lactulose |
|  | NDC | 00121457706 | Lactulose |
|  | NDC | 00527512078 | Lactulose |
|  | NDC | 00121115406 | Lactulose |
|  | NDC | 00472135832 | Lactulose |
|  | NDC | 00121115400 | Lactulose |
|  | NDC | 00182607558 | Lactulose |
|  | NDC | 50383079516 | Lactulose |
|  | NDC | 62794050117 | Lactulose |
|  | NDC | 60432003864 | Lactulose |
|  | NDC | 00591251938 | Lactulose |
|  | NDC | 50383077908 | Lactulose |
|  | NDC | 62559550103 | Lactulose |
|  | NDC | 50383077933 | Lactulose |
|  | NDC | 39328054108 | Lactulose |
|  | NDC | 42769134006 | Lactulose |
|  | NDC | 69067001015 | Lactulose |
|  | NDC | 45963043864 | Lactulose |
|  | NDC | 50383077916 | Lactulose |
|  | NDC | 00527512070 | Lactulose |
|  | NDC | 60432003732 | Lactulose |
|  | NDC | 60505036002 | Lactulose |
|  | NDC | 00527512578 | Lactulose |
|  | NDC | 62559550106 | Lactulose |
|  | NDC | 68115062608 | Lactulose |
|  | NDC | 00603137858 | Lactulose |
|  | NDC | 62135000337 | Lactulose |
|  | NDC | 00182607240 | Lactulose |
|  | NDC | 13668057412 | Lactulose |
|  | NDC | 00527512570 | Lactulose |
|  | NDC | 00472135808 | Lactulose |
|  | NDC | 60505036000 | Lactulose |
|  | NDC | 39328054116 | Lactulose |
|  | NDC | 00121457735 | Lactulose |
|  | NDC | 50383077931 | Lactulose |
|  | NDC | 54868310102 | Lactulose |
|  | NDC | 00472500060 | Lactulose |
|  | NDC | 00472136064 | Lactulose |
|  | NDC | 00121457730 | Lactulose |
|  | NDC | 00121115430 | Lactulose |
|  | NDC | 00121087332 | Lactulose |
|  | NDC | 66689003801 | Lactulose |
|  | NDC | 00121057730 | Lactulose |
|  | NDC | 13668058010 | Lactulose |
|  | NDC | 60505056202 | Lactulose |
|  | NDC | 62794050193 | Lactulose |
|  | NDC | 00121457715 | Lactulose |
| **Rifaximin** | NDC | 54868615401 | Rifaximin |
|  | NDC | 65649030105 | Rifaximin |
|  | NDC | 65649030302 | Rifaximin |
|  | NDC | 58016482401 | Rifaximin |
|  | NDC | 65649030304 | Rifaximin |
|  | NDC | 54868597200 | Rifaximin |
|  | NDC | 65649030103 | Rifaximin |
|  | NDC | 65649030141 | Rifaximin |
|  | NDC | 54868615402 | Rifaximin |
|  | NDC | 75839010600 | Rifaximin |
|  | NDC | 65649030303 | Rifaximin |
| **Ursodeoxycholic acid** | NDC | 00023614501 | Ursodiol |
|  | NDC | 00093536001 | Ursodiol |
|  | NDC | 00093536101 | Ursodiol |
|  | NDC | 00093938001 | Ursodiol |
|  | NDC | 00115152401 | Ursodiol |
|  | NDC | 00115152501 | Ursodiol |
|  | NDC | 00115993301 | Ursodiol |
|  | NDC | 00115993401 | Ursodiol |
|  | NDC | 00378173001 | Ursodiol |
|  | NDC | 00527132601 | Ursodiol |
|  | NDC | 00591236801 | Ursodiol |
|  | NDC | 00591236901 | Ursodiol |
|  | NDC | 00591299801 | Ursodiol |
|  | NDC | 00591300501 | Ursodiol |
|  | NDC | 00591315901 | Ursodiol |
|  | NDC | 00904622106 | Ursodiol |
|  | NDC | 00904622160 | Ursodiol |
|  | NDC | 00904622161 | Ursodiol |
|  | NDC | 00904648360 | Ursodiol |
|  | NDC | 00904689004 | Ursodiol |
|  | NDC | 10135054301 | Ursodiol |
|  | NDC | 10702023701 | Ursodiol |
|  | NDC | 12280030230 | Ursodiol |
|  | NDC | 12280030260 | Ursodiol |
|  | NDC | 24658078001 | Ursodiol |
|  | NDC | 38779198704 | Ursodiol |
|  | NDC | 38779198705 | Ursodiol |
|  | NDC | 38779198708 | Ursodiol |
|  | NDC | 42291085010 | Ursodiol |
|  | NDC | 42291085018 | Ursodiol |
|  | NDC | 42385094601 | Ursodiol |
|  | NDC | 42806050301 | Ursodiol |
|  | NDC | 43063087101 | Ursodiol |
|  | NDC | 43063087160 | Ursodiol |
|  | NDC | 43063087193 | Ursodiol |
|  | NDC | 49452808501 | Ursodiol |
|  | NDC | 49452808502 | Ursodiol |
|  | NDC | 49884041201 | Ursodiol |
|  | NDC | 49884041205 | Ursodiol |
|  | NDC | 49884041301 | Ursodiol |
|  | NDC | 49884041305 | Ursodiol |
|  | NDC | 50268079611 | Ursodiol |
|  | NDC | 50268079615 | Ursodiol |
|  | NDC | 50268079711 | Ursodiol |
|  | NDC | 50268079715 | Ursodiol |
|  | NDC | 51079038301 | Ursodiol |
|  | NDC | 51079038320 | Ursodiol |
|  | NDC | 51079097001 | Ursodiol |
|  | NDC | 51079097020 | Ursodiol |
|  | NDC | 51224015150 | Ursodiol |
|  | NDC | 51552090604 | Ursodiol |
|  | NDC | 51552090605 | Ursodiol |
|  | NDC | 51552090606 | Ursodiol |
|  | NDC | 51927290900 | Ursodiol |
|  | NDC | 52152006002 | Ursodiol |
|  | NDC | 52152006003 | Ursodiol |
|  | NDC | 52372063303 | Ursodiol |
|  | NDC | 52544093001 | Ursodiol |
|  | NDC | 55154335500 | Ursodiol |
|  | NDC | 58914078510 | Ursodiol |
|  | NDC | 58914078550 | Ursodiol |
|  | NDC | 58914079001 | Ursodiol |
|  | NDC | 58914079010 | Ursodiol |
|  | NDC | 58914079050 | Ursodiol |
|  | NDC | 60429013801 | Ursodiol |
|  | NDC | 60429066201 | Ursodiol |
|  | NDC | 60687010001 | Ursodiol |
|  | NDC | 60687010011 | Ursodiol |
|  | NDC | 60687037825 | Ursodiol |
|  | NDC | 60687037895 | Ursodiol |
|  | NDC | 60687052711 | Ursodiol |
|  | NDC | 60687052721 | Ursodiol |
|  | NDC | 62991218501 | Ursodiol |
|  | NDC | 62991218503 | Ursodiol |
|  | NDC | 62991218506 | Ursodiol |
|  | NDC | 62991279101 | Ursodiol |
|  | NDC | 62991279102 | Ursodiol |
|  | NDC | 62991279103 | Ursodiol |
|  | NDC | 63370032525 | Ursodiol |
|  | NDC | 63629104801 | Ursodiol |
|  | NDC | 63629117301 | Ursodiol |
|  | NDC | 63629228601 | Ursodiol |
|  | NDC | 63629228701 | Ursodiol |
|  | NDC | 63629835301 | Ursodiol |
|  | NDC | 64380084404 | Ursodiol |
|  | NDC | 64380084406 | Ursodiol |
|  | NDC | 64380084407 | Ursodiol |
|  | NDC | 64380091806 | Ursodiol |
|  | NDC | 64380091906 | Ursodiol |
|  | NDC | 64980013901 | Ursodiol |
|  | NDC | 64980048301 | Ursodiol |
|  | NDC | 64980048303 | Ursodiol |
|  | NDC | 64980048350 | Ursodiol |
|  | NDC | 66993040502 | Ursodiol |
|  | NDC | 66993040602 | Ursodiol |
|  | NDC | 68001022600 | Ursodiol |
|  | NDC | 68001022700 | Ursodiol |
|  | NDC | 68001034300 | Ursodiol |
|  | NDC | 68001034400 | Ursodiol |
|  | NDC | 68001037700 | Ursodiol |
|  | NDC | 68001037800 | Ursodiol |
|  | NDC | 68084021301 | Ursodiol |
|  | NDC | 68084021311 | Ursodiol |
|  | NDC | 68084089225 | Ursodiol |
|  | NDC | 68084089295 | Ursodiol |
|  | NDC | 68462047301 | Ursodiol |
|  | NDC | 68462047305 | Ursodiol |
|  | NDC | 68462047330 | Ursodiol |
|  | NDC | 68462047401 | Ursodiol |
|  | NDC | 68462047405 | Ursodiol |
|  | NDC | 68462047430 | Ursodiol |
|  | NDC | 69238154001 | Ursodiol |
|  | NDC | 70710112701 | Ursodiol |
|  | NDC | 70710112705 | Ursodiol |
|  | NDC | 70710112801 | Ursodiol |
|  | NDC | 70710112805 | Ursodiol |
|  | NDC | 70710148301 | Ursodiol |
|  | NDC | 70771140701 | Ursodiol |
|  | NDC | 70771140705 | Ursodiol |
|  | NDC | 70771140801 | Ursodiol |
|  | NDC | 70771140805 | Ursodiol |
|  | NDC | 70771152401 | Ursodiol |
|  | NDC | 71930001512 | Ursodiol |
|  | NDC | 72887014306 | Ursodiol |
|  | NDC | 72887014406 | Ursodiol |
|  | NDC | 79739714301 | Ursodiol |
|  | NDC | 79739714401 | Ursodiol |
|  | NDC | 80056014301 | Ursodiol |
|  | NDC | 80056014401 | Ursodiol |
| **Liver transplant - outcome** | ICD-10 pcs | 0FY00Z1 | Transplantation of liver, syngeneic, open approach |
|  | ICD-10 pcs | 0FY00Z0 | Transplantation of liver, allogeneic, open pproach |
|  | HCPCS/CPT-4 | 47135 | Liver allotransplantation; orthotopic; partial or whole, from cadaver or living donor, any age |
| **Gastric bypass procedure - attrition/exclusion** | HCPCS/CPT-4 | 48345 | Gastric bypass |
|  | HCPCS/CPT-4 | 43846 | Gastric bypass |
|  | HCPCS/CPT-4 | 43847 | Gastric bypass |
|  | HCPCS/CPT-4 | 43644 | Laparoscopic gastric bypass |
|  | HCPCS/CPT-4 | 43645 | Laparoscopic gastric bypass |
| **Not used** | HCPCS/CPT-4 | 43770 | Gastric lap band |
| **Gastric bypass procedure - attrition/exclusion** | ICD-10 pcs | 0DTB0ZZ | Resection of ileum, open approach |
|  | ICD-10 pcs | 0DTB4ZZ | Resection of ileum, percutaneous endoscopic approach |
|  | ICD-10 pcs | 0DTB7ZZ | Resection of ileum, via natural or artificial opening |
|  | ICD-10 pcs | 0DTB8ZZ | Resection of ileum, via natural or artificial opening endoscopic |
| **Livery biopsy procedure - demos (baseline compensated cirrhosis** | HCPCS/CPT-4 | 47000 |  |
|  | HCPCS/CPT-4 | 47001 |  |
|  | HCPCS/CPT-4 | 47100 |  |
|  | ICD-10 pcs | 0FB00ZX |  |
|  | ICD-10 pcs | 0FB03ZX |  |
|  | ICD-10 pcs | 0FB04ZX |  |
|  | ICD-10 pcs | 0FB10ZX |  |
|  | ICD-10 pcs | 0FB13ZX |  |
|  | ICD-10 pcs | 0FB14ZX |  |
|  | ICD-10 pcs | 0FB20ZX |  |
|  | ICD-10 pcs | 0FB23ZX |  |
|  | ICD-10 pcs | 0FB24ZX |  |
|  | ICD-10 pcs | 5012 |  |
|  | ICD-10 pcs | 5011 |  |
|  | ICD-10 pcs | 5014 |  |
|  | HCPCS/CPT-4 | 91200 |  |
|  | HCPCS/CPT-4 | 76700 |  |
|  | HCPCS/CPT-4 | 76705 |  |
|  | ICD-10 pcs | BF45ZZZ |  |
|  | ICD-10 pcs | 8874 |  |
|  | HCPCS/CPT-4 | 74181 |  |
|  | HCPCS/CPT-4 | 74182 |  |
|  | HCPCS/CPT-4 | 74183 |  |
|  | HCPCS/CPT-4 | 74185 |  |
|  | ICD-10 pcs | BF35Y0Z |  |
|  | ICD-10 pcs | BF35YZZ |  |
|  | ICD-10 pcs | BF35ZZZ |  |
|  | HCPCS/CPT-4 | 74150 |  |
|  | HCPCS/CPT-4 | 74160 |  |
|  | HCPCS/CPT-4 | 74170 |  |
|  | ICD-10 pcs | BF2500Z |  |
|  | ICD-10 pcs | BF250ZZ |  |
|  | ICD-10 pcs | BF2510Z |  |
|  | ICD-10 pcs | BF251ZZ |  |
|  | ICD-10 pcs | BF25Y0Z |  |
|  | ICD-10 pcs | BF25YZZ |  |
|  | ICD-10 pcs | BF25ZZZ |  |
|  | ICD-10 pcs | BF2600Z |  |
|  | ICD-10 pcs | BF260ZZ |  |
|  | ICD-10 pcs | BF2610Z |  |
|  | ICD-10 pcs | BF261ZZ |  |
|  | ICD-10 pcs | BF26Y0Z |  |
|  | ICD-10 pcs | BF26YZZ |  |
|  | ICD-10 pcs | BF26ZZZ |  |
|  | ICD-10 pcs | BF2C00Z |  |
|  | ICD-10 pcs | BF2C0ZZ |  |
|  | ICD-10 pcs | BF2C10Z |  |
|  | ICD-10 pcs | BF2C1ZZ |  |
|  | ICD-10 pcs | BF2CY0Z |  |
|  | ICD-10 pcs | BF2CYZZ |  |
|  | ICD-10 pcs | BF2CZZZ |  |
|  | ICD-10 pcs | BW2000Z |  |
|  | ICD-10 pcs | BW200ZZ |  |
|  | ICD-10 pcs | BW2010Z |  |
|  | ICD-10 pcs | BW201ZZ |  |
|  | ICD-10 pcs | BW20Y0Z |  |
|  | ICD-10 pcs | BW20YZZ |  |
|  | ICD-10 pcs | BW20ZZZ |  |

Abbreviations: CPT, Current Procedural Terminology; HCPCS, Healthcare Common Procedure Coding System; HCTZ, Hydrochlorothiazide; ICD-9/10, *International Classification of Diseases, Ninth/Tenth Revision*; NDC, National Drug Code; OCA, obeticholic acid; pcs, procedure coding system.

**Supplemental Table 2.** Original Cox Proportional Model With Individual Uncontrolled ALP and Uncontrolled Bilirubin (N=2370)

| **Outcomes** | **Parameters** | **Unadjusted** | | | | **Adjusted** | | | |
| --- | --- | --- | --- | --- | --- | --- | --- | --- | --- |
|  |  | **Hazard Ratio** | **Lower CL** | **Upper CL** | ***P* Value** | **Hazard Ratio** | **Lower CL** | **Upper CL** | ***P* Value** |
| Composite endpoint | Proportion of uncontrolled ALP time (≥1×ULN) (per 10% increase) | 1.059 | 1.034 | 1.086 | <0.001 | 1.065 | 1.039 | 1.092 | <0.001 |
|  | Proportion of uncontrolled ALP time (≥1.2×ULN) (per 10% increase) | 1.071 | 1.047 | 1.097 | <0.001 | 1.077 | 1.052 | 1.103 | <0.001 |
|  | Proportion of uncontrolled ALP time (≥1.5×ULN) (per 10% increase) | 1.092 | 1.066 | 1.119 | <0.001 | 1.104 | 1.077 | 1.131 | <0.001 |
|  | Proportion of uncontrolled ALP time (≥1.67×ULN) (per 10% increase) | 1.099 | 1.072 | 1.127 | <0.001 | 1.113 | 1.085 | 1.142 | <0.001 |
|  | Proportion of uncontrolled ALP time (≥2×ULN) (per 10% increase) | 1.116 | 1.085 | 1.148 | <0.001 | 1.135 | 1.103 | 1.168 | <0.001 |
| Composite endpoint | Proportion of uncontrolled bilirubin time (≥0.6×ULN) (per 10% increase) | 1.115 | 1.088 | 1.143 | <0.001 | 1.092 | 1.065 | 1.120 | <0.001 |
|  | Proportion of uncontrolled bilirubin time (≥0.8×ULN) (per 10% increase) | 1.161 | 1.133 | 1.191 | <0.001 | 1.139 | 1.110 | 1.169 | <0.001 |
|  | Proportion of uncontrolled bilirubin time (≥1×ULN) (per 10% increase) | 1.178 | 1.145 | 1.211 | <0.001 | 1.157 | 1.123 | 1.191 | <0.001 |
|  | Proportion of uncontrolled bilirubin time (≥2×ULN) (per 10% increase) | 1.371 | 1.296 | 1.451 | <0.001 | 1.374 | 1.289 | 1.464 | <0.001 |

Abbreviations: ALP, alkaline phosphatase; CL, confidence level; ULN, upper limit of normal.

**Supplemental Table 3.** Sensitivity Analysis #1 (Cox Proportional Model Among Only Patients With a Confirmed PBC Diagnosis Based on ICD-10 Codes [N=1532])

| **Parameter** | **Hazard Ratio** | **Lower CL** | **Upper CL** | ***P* Value** |
| --- | --- | --- | --- | --- |
| Proportion of uncontrolled ALP time (≥1×ULN) (per 10% increase) | 1.080 | 1.034 | 1.129 | <0.001 |
| Proportion of uncontrolled bilirubin time (≥0.6×ULN) (per 10% increase) | 1.075 | 1.027 | 1.125 | <0.001 |
| Age (per 10-year increase) | 2.146 | 1.724 | 2.671 | <0.001 |
| Sex (female vs male) | 0.626 | 0.391 | 1.003 | 0.052 |
| NASH (yes vs no) | 1.340 | 0.671 | 2.673 | 0.407 |
| Cirrhosis (yes vs no) | 1.381 | 0.821 | 2.323 | 0.223 |

Abbreviations: ALP, alkaline phosphatase; CL, confidence level; ICD-10, *International Classification of Diseases, Tenth Revision*; NASH, nonalcoholic steatohepatitis; PBC, primary biliary cholangitis; ULN, upper limit of normal.

**Supplemental Table 4.** Sensitivity Analysis #1 (Cox Proportional Model With Individual Uncontrolled ALP and Uncontrolled Bilirubin Among Only Patients With a Confirmed PBC Diagnosis Based on ICD-10 Codes [N=1532])

| **Outcomes** | **Parameters** | **Unadjusted** | | | | **Adjusted** | | | |
| --- | --- | --- | --- | --- | --- | --- | --- | --- | --- |
|  |  | **Hazard Ratio** | **Lower CL** | **Upper CL** | ***P* Value** | **Hazard Ratio** | **Lower CL** | **Upper CL** | ***P* Value** |
| Composite endpoint | Proportion of uncontrolled ALP time (≥1×ULN) (per 10% increase) | 1.079 | 1.033 | 1.126 | <0.001 | 1.086 | 1.039 | 1.134 | <0.001 |
|  | Proportion of uncontrolled ALP time (≥1.2×ULN) (per 10% increase) | 1.096 | 1.053 | 1.142 | <0.001 | 1.110 | 1.065 | 1.157 | <0.001 |
|  | Proportion of uncontrolled ALP time (≥1.5×ULN) (per 10% increase) | 1.126 | 1.080 | 1.174 | <0.001 | 1.142 | 1.095 | 1.191 | <0.001 |
|  | Proportion of uncontrolled ALP time (≥1.67×ULN) (per 10% increase) | 1.148 | 1.101 | 1.198 | <0.001 | 1.156 | 1.108 | 1.206 | <0.001 |
|  | Proportion of uncontrolled ALP time (≥2×ULN) (per 10% increase) | 1.164 | 1.112 | 1.219 | <0.001 | 1.171 | 1.119 | 1.227 | <0.001 |
| Composite endpoint | Proportion of uncontrolled bilirubin time (≥0.6×ULN) (per 10% increase) | 1.114 | 1.067 | 1.164 | <0.001 | 1.081 | 1.033 | 1.131 | <0.001 |
|  | Proportion of uncontrolled bilirubin time (≥0.8×ULN) (per 10% increase) | 1.181 | 1.130 | 1.235 | <0.001 | 1.144 | 1.093 | 1.198 | <0.001 |
|  | Proportion of uncontrolled bilirubin time (≥1×ULN) (per 10% increase) | 1.224 | 1.161 | 1.290 | <0.001 | 1.180 | 1.117 | 1.247 | <0.001 |
|  | Proportion of uncontrolled bilirubin time (≥2×ULN) (per 10% increase) | 1.418 | 1.284 | 1.566 | <0.001 | 1.381 | 1.231 | 1.548 | <0.001 |

Abbreviations: ALP, alkaline phosphatase; CL, confidence level; ICD-10, *International Classification of Diseases, Tenth Revision*; PBC, primary biliary cholangitis; ULN, upper limit of normal.

**Supplemental Table 5.** Sensitivity Analysis #2 (Cox Proportional Model Among Patients Without NASH or Cirrhosis [N=1977])

| **Parameter** | **Hazard Ratio** | **Lower CL** | **Upper CL** | ***P* Value** |
| --- | --- | --- | --- | --- |
| Proportion of uncontrolled ALP time (≥1×ULN) (per 10% increase) | 1.054 | 1.024 | 1.084 | <0.001 |
| Proportion of uncontrolled bilirubin time (≥0.6×ULN) (per 10% increase) | 1.079 | 1.048 | 1.111 | <0.001 |
| Age (per 10-year increase) | 1.565 | 1.393 | 1.759 | <0.001 |
| Sex (female vs male) | 0.564 | 0.422 | 0.755 | <0.001 |

Abbreviations: ALP, alkaline phosphatase; CL, confidence level; NASH, nonalcoholic steatohepatitis; ULN, upper limit of normal.

**Supplemental Table 6.** Sensitivity Analysis #2 (Cox Proportional Model With Individual Uncontrolled ALP and Uncontrolled Bilirubin Among Patients Without NASH or Cirrhosis [N=1977])

| **Outcomes** | **Parameters** | **Unadjusted** | | | | **Adjusted** | | | |
| --- | --- | --- | --- | --- | --- | --- | --- | --- | --- |
|  |  | **Hazard Ratio** | **Lower CL** | **Upper CL** | ***P* Value** | **Hazard Ratio** | **Lower CL** | **Upper CL** | ***P* Value** |
| Composite endpoint | Proportion of uncontrolled ALP time (≥1×ULN) (per 10% increase) | 1.051 | 1.022 | 1.081 | <0.001 | 1.059 | 1.030 | 1.090 | <0.001 |
|  | Proportion of uncontrolled ALP time (≥1.2×ULN) (per 10% increase) | 1.064 | 1.035 | 1.092 | <0.001 | 1.070 | 1.041 | 1.099 | <0.001 |
|  | Proportion of uncontrolled ALP time (≥1.5×ULN) (per 10% increase) | 1.087 | 1.057 | 1.118 | <0.001 | 1.098 | 1.068 | 1.130 | <0.001 |
|  | Proportion of uncontrolled ALP time (≥1.67×ULN) (per 10% increase) | 1.095 | 1.063 | 1.128 | <0.001 | 1.108 | 1.076 | 1.141 | <0.001 |
|  | Proportion of uncontrolled ALP time (≥2×ULN) (per 10% increase) | 1.109 | 1.073 | 1.146 | <0.001 | 1.130 | 1.093 | 1.168 | <0.001 |
| Composite endpoint | Proportion of uncontrolled bilirubin time (≥0.6×ULN) (per 10% increase) | 1.109 | 1.078 | 1.140 | <0.001 | 1.083 | 1.052 | 1.115 | <0.001 |
|  | Proportion of uncontrolled bilirubin time (≥0.8×ULN) (per 10% increase) | 1.157 | 1.125 | 1.191 | <0.001 | 1.132 | 1.099 | 1.166 | <0.001 |
|  | Proportion of uncontrolled bilirubin time (≥1×ULN) (per 10% increase) | 1.168 | 1.130 | 1.208 | <0.001 | 1.149 | 1.110 | 1.189 | <0.001 |
|  | Proportion of uncontrolled bilirubin time (≥2×ULN) (per 10% increase) | 1.370 | 1.273 | 1.474 | <0.001 | 1.442 | 1.335 | 1.557 | <0.001 |

Abbreviations: ALP, alkaline phosphatase; CL, confidence level; NASH, nonalcoholic steatohepatitis; ULN, upper limit of normal.
